# Serum microRNAs targeting *ACE2* and *RAB14* genes distinguish asymptomatic from critical COVID-19 patients

**DOI:** 10.1016/j.omtn.2022.06.006

**Published:** 2022-06-11

**Authors:** Maria Calderon-Dominguez, Eva Trejo-Gutierrez, Almudena González-Rovira, Lucía Beltrán-Camacho, Marta Rojas-Torres, Sara Eslava-Alcón, Daniel Sanchez-Morillo, Juan Calderon-Dominguez, Mª Pilar Martinez-Nicolás, Estibaliz Gonzalez-Beitia, Mª Dolores Nieto-Martín, Teresa Trujillo-Soto, Manuel A. Rodríguez-Iglesias, Juan A. Moreno, Rafael Moreno-Luna, Mª Carmen Durán-Ruiz

**Affiliations:** 1 Biomedicine, Biotechnology and Public Health Department, Cádiz University, 11002 Cádiz, Spain; 2 Biomedical Research and Innovation Institute of Cadiz (INiBICA), 11009 Cádiz, Spain; 3 Biomedical Engineering and Telemedicine Research Group, Department of Automation Engineering, Electronics and Computer Architecture and Networks, Universidad de Cádiz, 11009 Cádiz, Spain; 4 Occupational Health Service, National Paraplegic Hospital, SESCAM, 45071 Toledo, Spain; 5 Internal Medicine Department, University Hospital Virgen del Rocío, Seville, Spain; 6 UGC Microbiología, University Hospital Puerta del Mar, Avda. Ana de Viya 21, 11009 Cádiz, Spain; 7 Cell Biology, Physiology and Immunology Department, Agrifood Campus of International Excellence (ceiA3), University of Cordoba, 14014 Córdoba, Spain; 8 Maimonides Biomedical Research Institute of Cordoba (IMIBIC), UGC Nephrology, Hospital Universitario Reina Sofia, 14004 Cordoba, Spain; 9 Laboratory of Neuroinflammation, National Paraplegic Hospital, SESCAM, 45071 Toledo, Spain

**Keywords:** MT: Non-coding RNAs, COVID-19, miRNA, biomarkers, *in silico* analysis, ACE2, TMPRSS2, RAB14

## Abstract

Despite the extraordinary advances achieved to beat COVID-19 disease, many questions remain unsolved, including the mechanisms of action of SARS-CoV-2 and which factors determine why individuals respond so differently to the viral infection. Herein, we performed an *in silico* analysis to identify host microRNA targeting *ACE2*, *TMPRSS2*, and/or RAB14, all genes known to participate in viral entry and replication. Next, the levels of six microRNA candidates previously linked to viral and respiratory-related pathologies were measured in the serum of COVID-19-negative controls (n = 16), IgG-positive COVID-19 asymptomatic individuals (n = 16), and critical COVID-19 patients (n = 17). Four of the peripheral microRNAs analyzed (hsa-miR-32-5p, hsa-miR-98-3p, hsa-miR-423-3p, and hsa-miR-1246) were upregulated in COVID-19 critical patients compared with COVID-19-negative controls. Moreover, hsa-miR-32-5p and hsa-miR-1246 levels were also altered in critical versus asymptomatic individuals. Furthermore, these microRNA target genes were related to viral infection, inflammatory response, and coagulation-related processes. In conclusion, SARS-CoV-2 promotes the alteration of microRNAs targeting the expression of key proteins for viral entry and replication, and these changes are associated with disease severity. The microRNAs identified could be taken as potential biomarkers of COVID-19 progression as well as candidates for future therapeutic approaches against this disease.

## Graphical abstract

**Figure:**
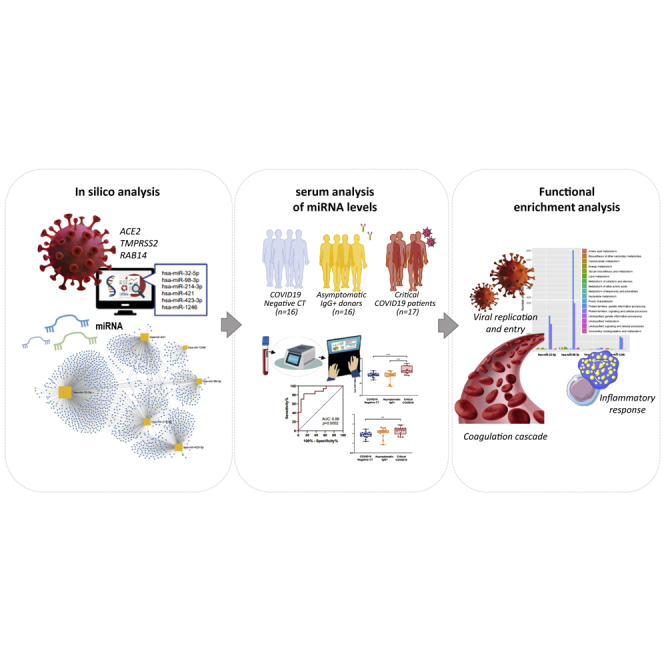

## Teaser

Herein, we have identified a panel of four miRNA targeting *ACE2* and *RAB14* genes significantly upregulated in the serum of COVID-19 critical patients compared with asymptomatic or negative individuals. These miRNAs might be taken as prognostic markers of SARS-CoV-2 infection, as well as potential therapeutic targets against COVID-19.

## Introduction

On 30 January, 2020, the World Health Organization declared coronavirus disease 19 (COVID-19) a public health emergency of international concern, becoming a global pandemic on 11 March, 2020.^1^ COVID-19 has drastically affected the entire world, at both economic and public health levels, with millions of deaths worldwide.^2^^,^^3^ The enormous effort in the development of vaccines against the severe acute respiratory syndrome coronavirus 2 (SARS-CoV-2) has significantly decreased the rhythm of disease progression. Unfortunately, it is still not possible to predict the efficacy and durability of immunity after vaccination, mainly taking into account the viral mutations constantly arising, as in the case of Omicron, one of the last variants identified.^4^^,^^5^ Thus, major efforts are actually focused on understanding the molecular mechanisms of SARS-CoV-2 infection, as well as on the identification of therapeutic targets against COVID-19 and potential biomarkers to prevent its progression.^6^ Two clear candidates in this therapeutic approach are angiotensin-converting enzyme 2 (ACE2), one of the main co-factors required by SARS-CoV-2 to access human host cells, and the transmembrane protease serine 2 (TMPRSS2), which is responsible for priming of the viral spike protein, a step required to allow the virus-host cell membrane fusion and further internalization of the virus.^7^^,^^8^ ACE2 and TMPRSS2 have received more attention; however, other host-cell proteins related to SARS-CoV-2 replication are also playing an essential role during viral infection, promoting its survival. For instance, the interaction of SARS-CoV-2 with host proteins involved in the assembly and viral trafficking, such as Ras-related protein Rab-14 (RAB14), may favor SARS-CoV-2 replication.^9^^,^^10^ Hence, the use of agents blocking or interfering with the interaction between SARS-CoV-2 and ACE2, TMPRSS2, or RAB14 might help to prevent or reduce the virus entry and replication. Indeed, some inhibitors are being tested. That is the case of camostat mesylate, targeting TMPRSS2, which seems to block SARS-CoV-2 infection of lung cells.^8^ Alternatively, the use of inhibitors against SARS-CoV-2 mRNA translation are also of interest.^11^^,^^12^ In this regard, the application of specific microRNAs (miRNAs) to regulate the expression of essential proteins for the SARS-CoV-2 infection process constitutes a promising but also unexplored approach.^13^^,^^14^

MiRNAs are small (18–22 nucleotides) highly conserved, non-coding single-stranded ribonucleic acids (RNAs), which appear to be involved in many physiological processes as well as in different diseases, participating, among others, in the modulation of viral infection and host defense.^15^ To date, several miRNAs have been found in altered levels in the blood of individuals after viral infection, including COVID-19 patients.^6^^,^^16^^,^^17^ Thus, miRNAs not only constitute promising therapeutic tools^17^ but they are also considered as potential prognostic markers for SARS-CoV-2 infection.^18^

In this study, based on the literature and *in silico* results, we focus on analyzing the levels of miRNA targeting *ACE2*, *TMPRSS2*, and *RAB14*, all associated with viral entry and replication in the serum of COVID-19 patients. Our results indicate that peripheral hsa-miR-32-5p, hsa-miR-98-3p, hsa-miR-423-3p, and has-miR-1246 increased in critical COVID-19 patients. The functional pathways in which these molecules participate, as well as the potential use of these molecules as biomarkers, are discussed.

## Results

### *In silico* analyses to predict miRNAs targeting *ACE2*, *TMPRSS2*, and *RAB14*

**Figure 1:**
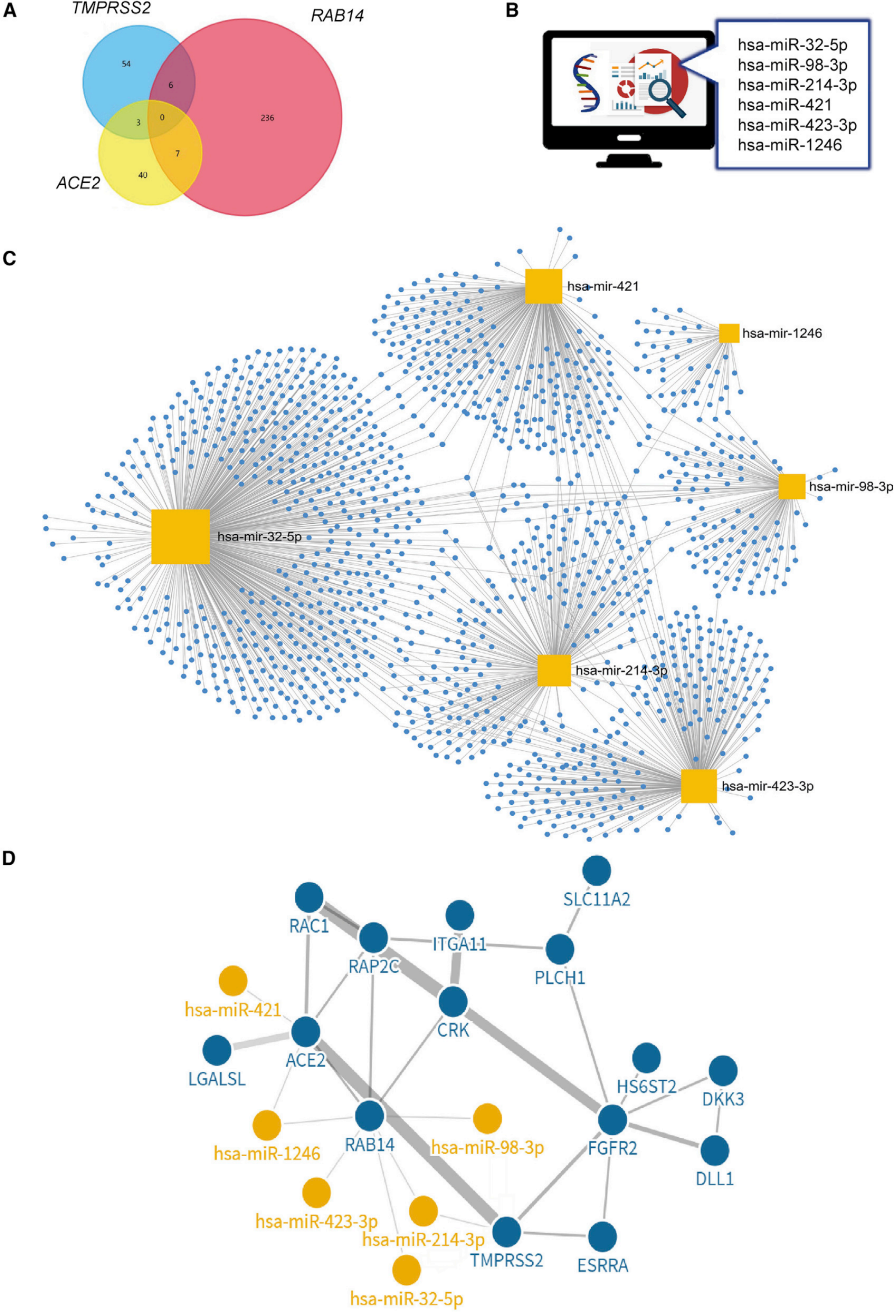
Bioinformatic analysis of the *ACE2*, *TMPRSS2*, and *RAB14* genes to predict their potential miRNAs (A) Venn diagram of the predicted miRNAs for *ACE2*, *TMPRSS*, and *RAB14* genes. (B) miRNAs related to the respiratory system were selected from bibliographic search. (C) The miRNA-mRNA network of selected miRNAs. (D) Interaction network of the shared target genes (blue) and the selected miRNAs (yellow). The size of the gray lines represents the degree of interaction between proteins.

The miRNAs potentially targeting *ACE2*, *TMPRSS2*, and/or *RAB14* genes were determined by *in silico* analysis using different miRNA target prediction tools (Figure 1A). Among all miRNA predicted to target these genes, only a few were related to the respiratory system or described as post-transcriptional regulators (Table S1). Finally, six miRNAs (hsa-miR-32-5p, hsa-miR-98-3p, hsa-miR-214-3p, hsa-miR-421, hsa-miR-423-3p, and hsa-miR-1246) were selected as our targets of interest (Figure 1B).

Next, the number of binding sites of all six miRNAs were analyzed, indicating the number of all selected miRNAs in the 3′ UTR of *ACE2*, *TMPRSS2*, and *RAB14* (Table S2). As previously described,^19^ a match of six nucleotides between miRNA and 3′ UTR sequence may be considered as a potential seeding region (Figure S1). hsa-miR-98-3p and hsa-miR-423-3p displayed the highest number, with six 3′ UTR binding sites for the *RAB14* gene.

Furthermore, the target genes of these six miRNA candidates were also predicted (Figure 1C). According to protein-protein interaction networks analysis of the shared target genes (Figure 1D), they all displayed a narrow interaction with each other (p = 0.003).

### Identification of differentially expressed miRNAs in critical COVID-19 patients

**Figure 2:**
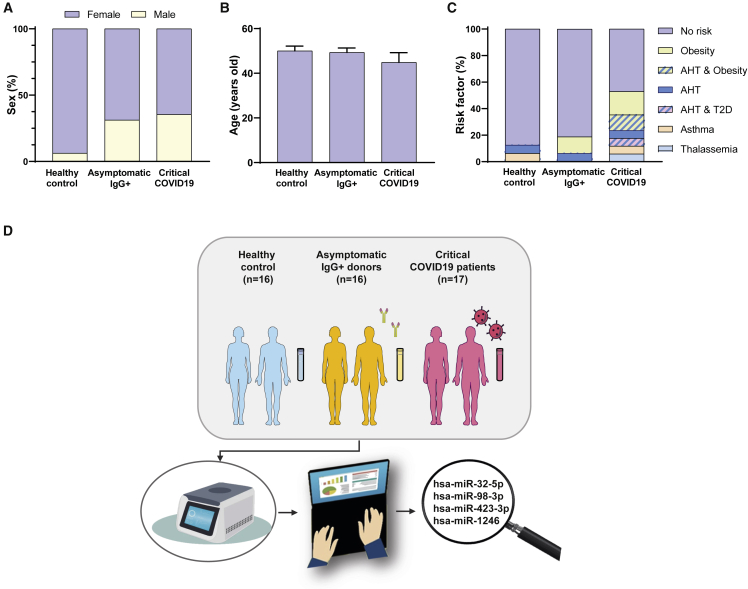
Study population and flow diagram of the experimental progress. Clinical variables of the study population: (A) Gender (%); (B) Age, represented as the mean +- SEM; (C) Risk factors found for individuals in each group (healthy controls, asymptomatic IgG+ individuals and critical COVID19 patients). (D) Flowchart of the study population and the protocol of the experimental steps, including qPCR analysis and the selected serum miRNAs.

Once the six miRNAs candidates were selected, the serum levels were analyzed by qPCR in our study population. A graphical representation of some characteristics registered for the study population is shown in Figure 2 (extended in Table S3). Most of the patients enrolled were female (Figure 2A). The mean ages for COVID-19-negative controls, asymptomatic IgG-positive subjects, and critical COVID-19 patients were 50 ± 2.18, 49.31 ± 1.99, and 44.88 ± 4.35 years, respectively (Figure 2B). Critical patients presented some risk factors, such as obesity (23.5%), arterial hypertension (AHT) (24%), diabetes mellitus type 2 (T2D) (5.88%), asthma (5.9%), and thalassemia (5.9%) (Figure 2C). Finally, the percentage of smokers in critical COVID-19 patients was 5.8%, 25% in COVID-19-negative controls, and 18.75% in asymptomatic individuals. A schematic representation of the study workflow is shown in Figure 2D.

**Figure 3:**
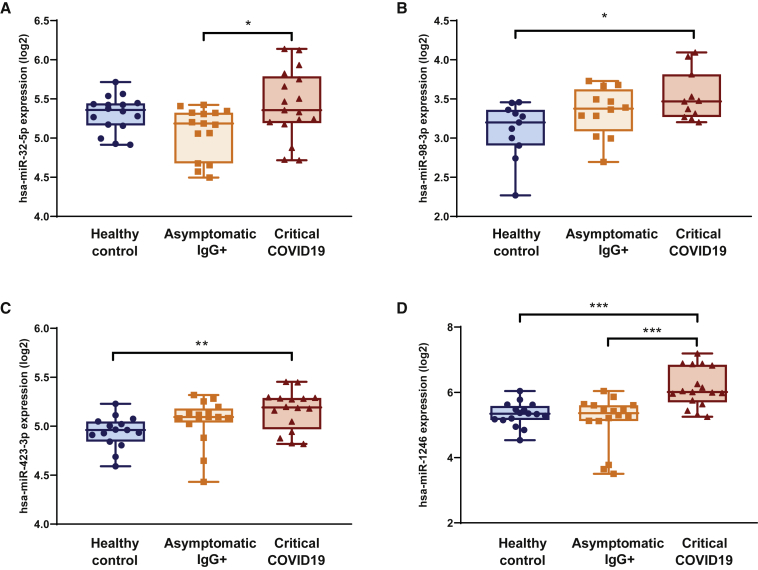
Serum miRNA levels, normalized to hsa-miR-103a-3p, in COVID-19-negative control subjects, asymptomatic IgG-positive donors, and critical COVID-19 patients (A) hsa-miR-32-5p, (B) hsa-miR-98-3p, (C) hsa-miR-423-3p, and (D) hsa-miR-1246 serum levels. Data are presented in log2 with box and plot graphs representing the median, min, and max values, showing all data points. ∗p < 0.05, ∗∗p < 0.01, ∗∗∗p < 0.001.

The miRNA levels detected in the serum samples are shown in Table 1. A total of four peripheral miRNAs (hsa-miR-32-5p, hsa-miR-98-3p, hsa-miR-423-3p, and hsa-miR-1246) were differentially expressed in our population (Figure 3). Thus, hsa-miR-98-3p, hsa-miR-423-3p, and hsa-miR-1246 were significantly increased in critical patients compared with COVID-19-negative subjects (Figures 3B–3D). Moreover, peripheral hsa-miR-32-5p and hsa-miR-1246 were differentially expressed in critical COVID-19 patients compared with asymptomatic IgG-positive donors (Figures 3A and 3D). In addition, according to the miRNA TissueAtlas platform, all four miRNAs have been found expressed in lungs, among other tissues (Figure S2).

**Table 1:** Peripheral miRNAs levels in the study groups

	COVID-19-negative control			Asymptomatic IgG-positive COVID-19				Critical COVID-19 patients					
	Median	Q1	Q3	Median	Q1		Q3	p value versus COVID-19-negative controls	Median	Q1	Q3	p value versus COVID-19-negative controls	p value versus aymptomatic IgG positive patients
hsa-miR-32-5p	5.359	5.161	5.444	5.186	4.677	5.324		0.1616	5.356	5.191	5.789	>0.9999	**0.0330**
hsa-miR-98-3p	3.201	2.905	3.362	3.377	3.087	3.624		0.2258	3.469	3.269	3.815	**0.0254**	>0.9999
hsa-miR-214-3p	3.869	3.737	4.125	3.793	3.534	4.164		>0.999	3.756	3.525	3.926	0.8096	>0.9999
hsa-miR-421	4.037	3.919	4.137	4.113	4.059	4.257		0.4034	4.148	4.074	4.361	0.1692	>0.9999
hsa-miR-423-3p	4.961	4.842	5.048	5.094	5.037	5.181		0.1207	5.192	4.969	5.289	**0.0048**	0.7860
hsa-miR-1246	5.343	5.155	5.583	5.361	5.121	5.605		>0.9999	6.009	5.695	6.846	**0.0007**	**0.0005**

Data presented as median (Q1-Q3). Coefficient significant at p < 0.05 (highlighed in bold).

### Diagnostic potential of serum miRNAs and the association with critical COVID-19 patients

**Figure 4:**
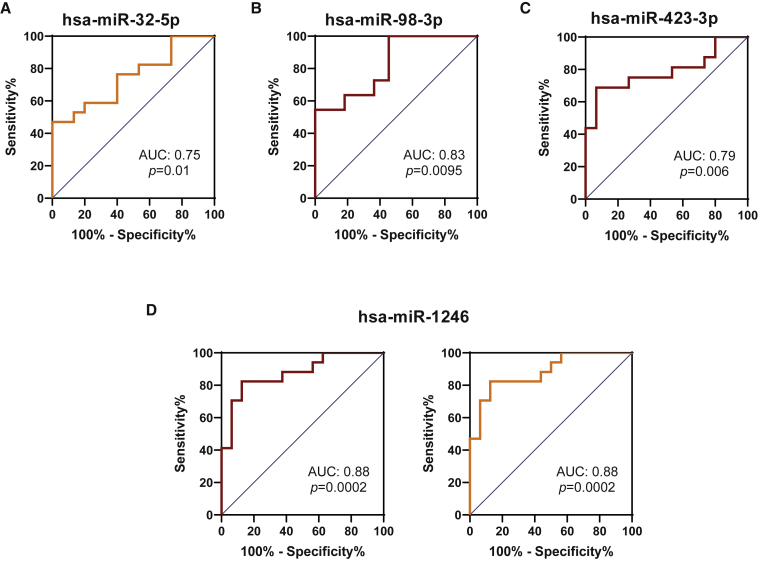
Receiver operating characteristic analysis of the miRNAs in critical COVID-19 patients with area under curve (A) Receiver operating characteristic (ROC) analysis of hsa-miR-32-p in critical COVID-19 versus asymptomatic IgG-positive individuals. (B) ROC analysis of hsa-miR-98-3p in critical COVID-19 compared with COVID-19-negative controls. (C) ROC analysis of hsa-miR-423-3p in critical COVID-19 compared with COVID-19-negative controls. (D) ROC analysis of hsa-miR-1246 in critical COVID-19 compared with COVID-19-negative controls (left panel) and compared with asymptomatic IgG-positive subjects (right panel).

Next, to analyze the diagnostic value, the area under the curve-receiver operating characteristic (AUC-ROC) was compared for the single and combined differentially expressed miRNAs (Table 2). Although the combined miRNA AUC values were significant, none of them reached a value higher than single miRNAs. The ROC curve of single hsa-miR-32-5p, hsa-miR-98-3p, hsa-miR-423-3p, and hsa-miR-1246 revealed the probability to use them as valuable biomarkers to diagnose critical COVID-19 patients from COVID-19-negative controls and asymptomatic IgG-positive individuals (Figure 4A-D). The highest discriminatory power achieved by a single miRNA was acquired for hsa-miR-1246, with an AUC of 0.875 (95% CI: 0.755–0.995; p = 0.0002) (Figure 4D).

**Table 2:** Comparisons of single and combined circulating miRNAs as predictors of critical COVID-19 patients

Groups	miRNA	AUC (95% CI)	Sensitivity (%)	Specificity (%)	p value
Critical COVID-19 versus COVID-19-negative control	hsa-miR-98-3p	0.8264 (0.6538–0.9991)	72.73	63.64	0.0095
	hsa-miR-423-3p	0.7875 (0.6199–0.9551)	75.00	73.33	0.0064
	hsa-miR-1246	0.8750 (0.755–0.995)	82.35	87.50	0.0002
	hsa-miR-98-3p + hsa-miR-423-3p + hsa-miR-1246	0.6634 (0.5484–0.7784)	63.64	69.05	0.0091
Critical COVID-19 versus asymptomatic IgG-positive COVID-19	hsa-miR-32-5p	0.7490 (0.5799–0.9181)	76.47	60.00	0.0165
	hsa-miR-1246	0.8824 (0.7681–0.9966)	82.35	87.50	0.0002
	hsa-miR-32-5p + hsa-miR-1246	0.7913 (0.6831–0.8994)	73.53	64.52	<0.0001

Finally, the association between peripheral miRNA levels and clinical variables was also evaluated (Table 3). The circulating hsa-miR-32-5p and hsa-miR1246 levels showed a negative correlation with the presence of risk factor. Furthermore, the circulating hsa-miR-32-5p levels indicated a positive correlation with the age and severity of COVID-19, which could be possible since miRNA expression is related to age.^20^ On the other hand, although a differential expression of miRNAs has been described between males and females, only the levels of hsa-98-3p showed an association with the gender.^21^

**Table 3:** Correlation between the individual miRNAs levels and clinical variables in critical COVID-19 patients

	Hsa-miR-32-5p		Hsa-miR-98-3p		Hsa-miR-423-3p		Hsa-miR-1246	
	Pearson r	p	Pearson r	p	Pearson r	p	Pearson r	p
Age	−0.594	**0.006**	0.323	0.166	0.393	0.066	−0.226	0.192
Sex	0.047	0.429	−0.655	**0.014**	0.056	0.418	−0.111	0.335
COVID-19 severity	0.414	**0.049**	0.065	0.425	−0.035	0.449	0.219	0.199
Risk factor	−0.506	**0.019**	−0.437	0.090	0.296	0.132	−0.565	**0.009**

### Functional enrichment analysis

**Figure 5:**
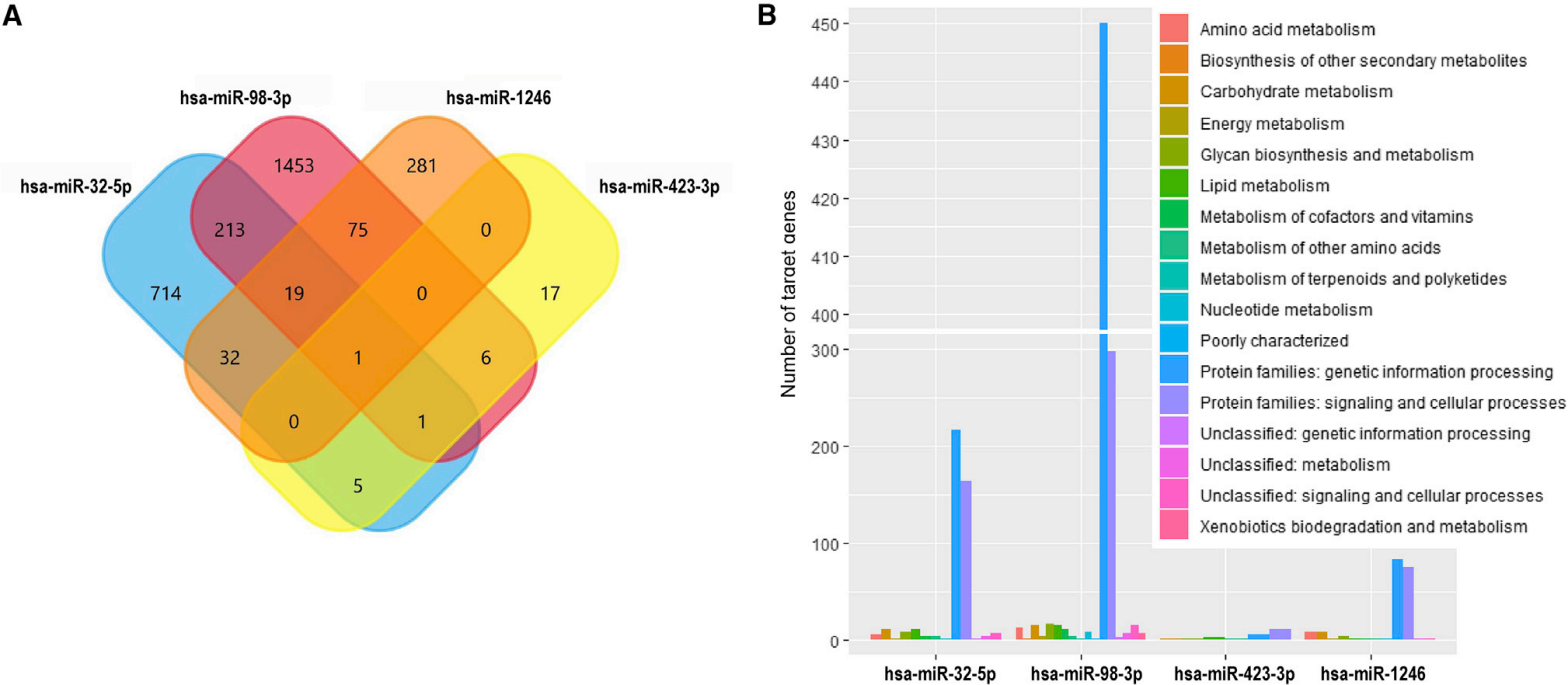
Target prediction of hsa-miR-32-5p, hsa-miR-98-3p, hsa-miR-423-3p, and hsa-miR-1246 (A) Venn diagram indicating the numbers of common and exclusive genes targeted by hsa-miR-32-5p, hsa-miR-98-3p, hsa-miR-423-3p, and hsa-miR-1246. B) KEGG categorization of targeted genes of hsa-miR-32-5p, hsa-miR-98-3p, hsa-miR-423-3p, and hsa-miR-1246; the x axis indicates KEGG categories, and the y axis indicates the numbers of gene targets.

A total of 2,812 genes were detected as potential targets of our four differentially quantified serum miRNAs. Remarkably, *RAB14* was the only common target gene for hsa-miR-32-5p, hsa-miR-98-3p, hsa-miR-423-3p, and hsa-miR-1246 (Figure 5A). According to the functional analysis performed via the KEGG database (Figure 5B), the genes targeted by these four miRNAs were mainly involved in the processing of genetic information, cell signaling, and other cell-related processes (extended in Figure S3).

**Figure 6:**
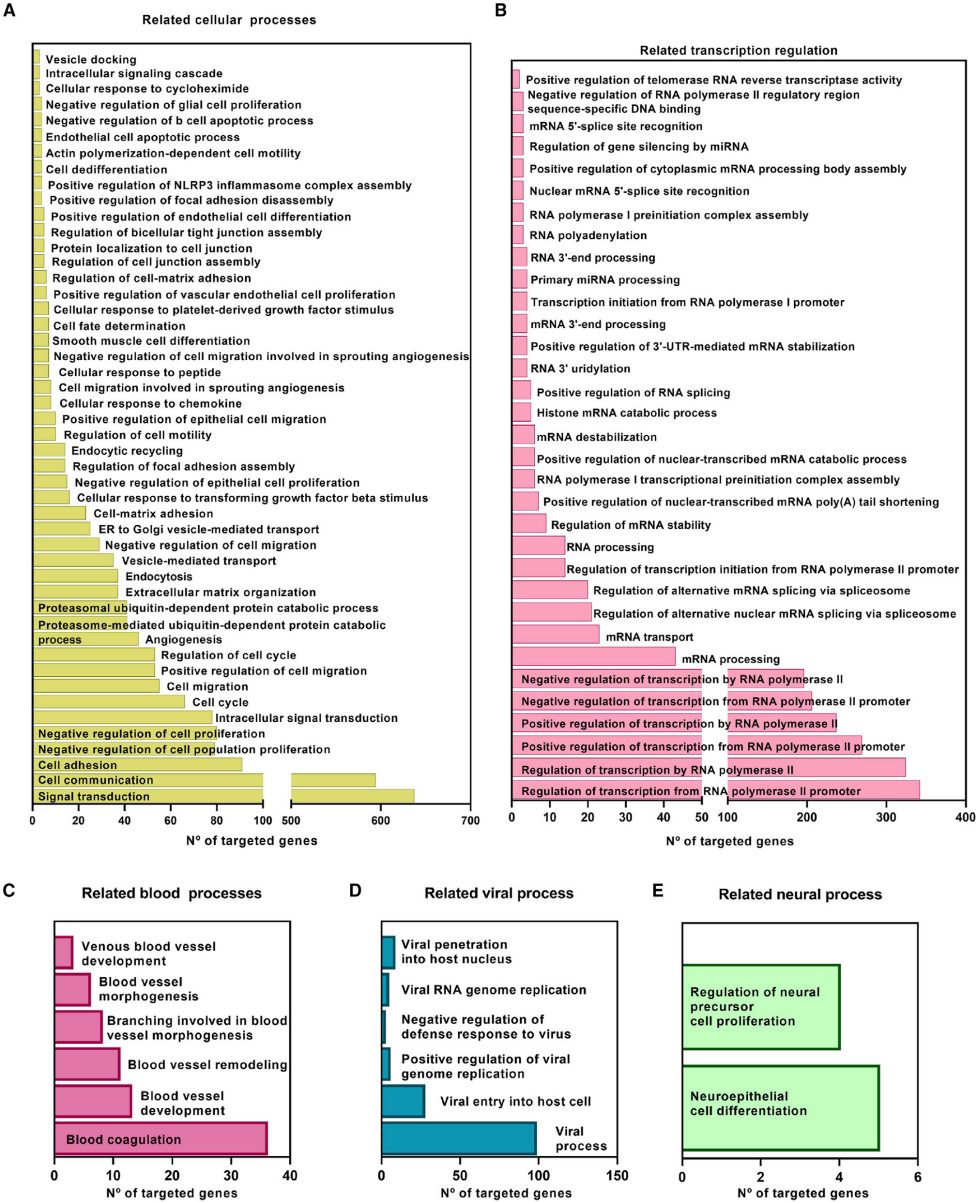
Functional enrichment analysis of hsa-miR-32-5p, hsa-miR-98-3p, hsa-miR-423-3p, and hsa-miR-1246 targeted genes Bar plots representing functional enrichment analysis of cellular-related (A), transcriptional-related (B), blood-related (C), viral-related (D), and neural-related (E) processes.

Moreover, the targeted genes were involved in several biological processes (Figures 6A–6E), which were all significantly enriched (extended in Table S4). Within the cellular process category, many genes correlated with vesicle trafficking and cell-cell interactions, as well as regulation of cell cycle, cell proliferation, migration, or even regulation of the NLRP3 inflammasome complex (Figure 6A). Many genes were involved in RNA transcription and mRNA processing and transport, which correlates with the involvement of miRNAs in the regulation of RNA polymerase I and II expression, mRNA processing, stability, and splicing, among others (Figure 6B). Moreover, these genes were also directly linked to the viral process, including viral RNA replication and viral entry into the host nucleus or regulation of the viral genome replication (Figure 6D). Also, all four miRNAs have been previously associated to respiratory-related diseases (extended in Table S5). Finally, several genes were associated to blood-related processes, such as coagulation, vessel development, morphogenesis, and remodeling (Figure 6C), and also to neuroepithelial cell differentiation and regulation of neural precursor cell proliferation, among others (Figures 6D and 6E).

## Discussion

After 2 years since the first person was diagnosed with COVID-19, more than 447 million cases have been reported around the world, including more than 6 million deaths.^22^ To date, numerous diagnostic methods and vaccines have been developed in record time, thanks to extraordinary and unprecedented scientific and clinical efforts. Nevertheless, the mechanisms of action of SARS-CoV-2, as well as the potential secondary effects that this virus exerts over the organism are not fully understood yet.

One of the unanswered questions regarding COVID-19 is why some people infected with SARS-CoV-2 present severe symptoms, even though others do not. Most patients show mild to moderate symptoms such as fever, persistent dry cough, body aches, and occasional dyspnea. However, a small fraction of patients may also present acute respiratory failure and acute respiratory distress syndrome associated with multiple organ failure.^23^^,^^24^ Consequently, knowing an individual’s susceptibility to SARS-CoV-2 infection, through identifying critical biomarkers, may guide future treatment strategies for the disease and its different phases. In this sense, peripheral miRNAs have been remarked as potential biomarkers for COVID-19 treatment and diagnosis since they can play an essential role in the pathogenesis of its infection.^6^^,^^25^

In this study, we focused on the identification of miRNAs targeting *ACE2*, *TMPRSS2*, and *RAB14* genes, due to their direct or indirect association with SARS-CoV-2 infection. While other miRNAs had been recently associated with the pathology of COVID-19, being upregulated in acute and critical patients compared with controls (i.e., miR-29a-3p, -146a-3p, -155-5p,^25^ or miR-6501-5p and miR-618^6^), none of them targeted *ACE2*, *TMPRSS2*, or *RAB14*. Notably, four of the six miRNAs selected from the *in silico* analysis (hsa-miR-32-5p, hsa-miR-98-3p, hsa-miR-423-3p, and hsa-miR-1246) were significantly increased in the serum of critical COVID-19 patients. In addition, hsa-miR1246 could discriminate between IgG-positive asymptomatic subjects and critical COVID-19 patients. Furthermore, based on their AUC-ROC values, these four miRNAs could be considered novel biomarkers with high-yield diagnostic accuracy. In addition, hsa-miR-32-5p and hsa-miR-1246 correlated with the presence of obesity,^26^^,^^27^ AHT,^28^ T2D,^29^ asthma,^30^ or thalassemia,^31^ which have been described as risk factors for critical COVID-19 patients.

Remarkably, none of the four miRNAs found altered in the serum of critical COVID-19 patients had *TMPRSS2* as a target gene, while they all targeted *RAB14*. Among them, hsa-miR-32-5p has been associated *in silico* to *RAB14* in pancreatic and colon tumors.^32^ Moreover, hsa-miR-32-5p and miR-98-3p, also targeting *RAB14*, were identified as crucial lung cancer-associated miRNAs,^33^, ^34^, ^35^ and their deregulation levels were identified in several respiratory disorders, such as acute respiratory distress syndrome,^36^^,^^37^ bronchopulmonary dysplasia,^38^ and congenital pulmonary airway malformations.^39^ Besides, these miRNAs have been previously related with viral processes. For instance, miR-32-5p has a significant regulatory role in avian hepatitis A viral infection,^40^ visna maedi virus,^41^ and caprine arthritis encephalitis viruses,^41^ while the deregulation of the expression miR-98 has been described in a mouse model of West Nile virus neuropathogenesis.^42^

Apart from these two miRNAs, hsa-miR-423-3p also exhibited *RAB14* as a unique target gene. While previous studies found increased levels of the circulating 5′ form (hsa-miR-423-5p) in COVID-19 patients, nothing was described concerning hsa-miR-423-3p.^43^ This miRNA has been defined as a biomarker of lung cancer,^44^ and it has also been related to tuberculosis and endocytosis pathways, in which the RAB protein family plays a crucial role.^45^

Finally, according to our *in silico* analysis, hsa-miR-1246 was the only miRNA that targeted both *ACE2* and *RAB14* genes. As mentioned above, hsa-miR-1246 not only discriminated critical COVID-19 patients versus COVID-19-negative controls, it also allowed to distinguish critical versus asymptomatic patients, in agreement with a recent study in which has-miR-1246 appeared differentially expressed in severe versus asymptomatic COVID-19 patients, although this study was performed only with male patients.^46^ Notablhashsa-miR-1246 has been described as a biomarker of emphysema in patients with chronic obstructive pulmonary disease,^47^ and also with non-small cell lung cancer progression.^48^ Moreover, miR-1246 has been identified as a possible regulator of the SARS-CoV-2 genome, which would provide more information on the protection mechanisms associated with miRNAs.^49^ Concerning *ACE2* expression, a preliminary study described that the *ACE2* mRNA levels were inversely proportional to miR-1246 levels in the airways epithelium of smokers.^50^ Noteworthy, in our study population most of our critical COVID-19 patients were non-smokers. Moreover, Khan and co-workers described, both *in silico* and *in vitro*, that *ACE2* was regulated by miR-1246 in patients with acute respiratory distress syndrome.^51^^,^^52^ Finally, as regards *Rhas4*, hsa-miR-1246 has been also described as a potential prognostic biomarker for glioma, being predicted as one of its target genes.^53^

In addition to *ACE2* and *RAB14*, another 2,810 target genes were predicted for our 4 miRNAs. Overall, our *in silico* analysis indicated that these miRNAs are much more than simple post-transcriptional regulators since, besides, they have been involved in several biological processes related to the pathogenesis of SARS-CoV-2. For instance, the increase seen of these four circulating miRNAs may correlate, for example, with the decreased levels of *RAB14* transcripts found in lung biopsies from patients with adenocarcinomas,^54^ together with many other crucial genes for SARS-CoV-2 infection. RAB14 participates in the formation of vesicles^55^ necessary for the maturation and assembly of the structural proteins of SARS,^9^^,^^10^ therefore it might also constitute an essential protein for infection by SARS-CoV-2.^56^ In this sense, the analysis of the biological and functional roles indicated that not only *RAB14* but many other targeted genes appear involved in vesicular trafficking, cell-cell interactions, and regulation of the cell cycle, among others. In addition, these genes also participate in viral entry into the host cell, viral RNA replication, or even regulation of viral genome replication, which, together with the possible inhibition of the expression of RAB14 by the increase of the four miRNAs, could indicate a defense mechanism of the cells against the proliferation of the virus. Finally, several genes were also associated to regulation of the NLRP3 inflammasome complex, which has been linked to the severity of COVID-19,^57^ and also with blood- and coagulation-related processes. This might correlate with the hypercoagulability and thrombotic events that take place in response of COVID-19.^58^

### Conclusions

In this study, we have identified four miRNAs targeting *ACE2* and/or *RAB14* that could be taken as potential biomarkers of COVID-19 progression, allowing to distinguish critical patients from asymptomatic and negative individuals. The identified miRNA have been previously associated to respiratory-related diseases, including SARS-CoV. Moreover, many other gene targets of these miRNA have been associated with viral replication and inflammation- and coagulation-related processes. The individuals included in this study were recruited before being vaccinated, so any potential effect that vaccines might have over our results should be further evaluated. On the other hand, the limited access to serum samples in our study population constitutes a clear limitation in our study. Ideally, a higher number of samples should be analyzed to further validate the specificity of these miRNAs as biomarkers. Besides, even though peripheral miRNA levels were quantified, there was no confirmation about the direct secretion from the respiratory tissues into the extracellular space in COVID-19 patients, although previous research has shown their presence in the lungs. Future studies should include *in vitro* and *in vivo* models of COVID-19 to corroborate the bioinformatic predictions. These analyses might confirm the involvement of this four-miRNA panel as prognostic markers of SARS-CoV-2 infection, as well as their potential role as therapeutic candidates to inhibit the host response against this or other related viruses.

## Materials and methods

### Bioinformatic analysis to predict miRNAs that target ACE2, TMPRSS2, and RAB14

The miRDB database (http://mirdb.org/) and TargetScan (http://www.targetscan.org) were used to predict the miRNAs and their targeted genes,^19^^,^^59^ and the network image was obtained using the miRNet (https://www.mirnet.ca) tool.^60^ All these analyses were performed using the default parameters, and *Homo sapiens* was selected as the specific Taxonomy. The shared target genes of the predicted miRNAs were analyzed with STRING, an on-line platform to identify functional protein-association networks (https://string-db.org),^61^ while the miRNA-gene network image was obtained with Flourish software (https://flourish.studio). Finally, STarMiR (www.sfold.wadsworth.org/cgi-bin/starmir.pl) was used for the prediction of miRNA binding sites to 3′ UTR mRNA binding sites (seeding region).

### Study population

In total, 49 subjects were included in this study. Based on qPCR analysis against SARS-CoV-2, and ELISA tests for specific IgG and IgM antibodies (IME00136 and IME00137, Erba Mannheim), subjects were classified into three groups: (1) COVID-19-negative controls, which were PCR and IgG negative at the time of serum extraction (n = 16), (2) asymptomatic COVID-19 individuals, PCR negative and IgG positive at the time of serum extraction (n = 16), and (3) critical COVID-19 patients (n = 17) who required hospitalization. The first two groups (COVID-19-negative controls and asymptomatic donors) were enrolled at the National Paraplegic Hospital (Toledo, Spain), between April and May 2020. Critical COVID-19 patients were recruited at the time of hospitalization at the University Hospital Puerta del Mar (Cadiz, Spain) in July 2021, and the COVID-19 Hospital (Seville, Spain) in May 2021. The protocol was approved by the ethics committee at each center, and the study was conducted in accordance with the Helsinki II Declaration. Only donors older than 18 years were included in the study, and written informed consent was provided by all of those who participated in this study.

### Serum collection

Peripheral blood samples were collected with serum separator tubes (SST II Advance, BD Vacutainer). The blood was then mixed up and down 8–10 times. After that, SST tubes were incubated at room temperature for at least 30 min to ensure the separation of the serum from the cellular components, and the serum was collected by centrifugation (2,000 × *g* for 10 min at 4°C). Serum samples were aliquoted and stored at −80°C until further use.

### RNA isolation

Isolation of total RNA, including miRNAs, was performed with 200 μL of serum using the miRNeasy Serum/Plasma Kit (QIAGEN) following the manufacturer’s instructions. Before purification, each serum sample was spiked (RNA spike-in, QIAGEN) with UniSp2 (2 fmol), UniSp4 (0.02 fmol), UniSp5 (0.00002 fmol), and MS2 RNA (Merck) to monitor the technical quality of RNA isolation according to the manufacturer’s guidelines. Directly after isolation, RNA was subjected to the reverse transcription process.

### Reverse transcriptase reaction

An MiRCURY LNA Reverse Transcription (RT) Kit (QIAGEN) was used to synthesize cDNA according to manufacturer’s instructions. Isolated RNA (2 μL) were added to the reaction tube to make up a final volume of 10 μL reaction mix. UniSp6 (0.075 fmol) and cel-miR-39-3p (0.001 fmol) were used as positive controls for cDNA synthesis (QIAGEN). The reaction took place for 60 min at 42°C, heat inactivated for 5 min at 95°C, and immediately cooled to 4°C in a thermal cycler. Then cDNA samples were stored at −20°C.

### Real-time quantitative polymerase chain reaction analysis of miRNAs expression levels

Samples from the RT reaction were prepared with the miRCURY SYBR Green PCR Kit (QIAGEN) and assessed for miRNA gene expression using the miRCURY LNA miRNA Serum/Plasma Focus PCR Panels (QIAGEN) according to the manufacturer’s protocol. The interpolate calibrator UniSp3 was used to account for the variability between plates. Real-time qPCR analysis was performed in the CFX Connect PCR System (Bio-Rad) at 95°C for 2 min to heat samples, followed by 40 cycles of 95°C for 10 s, and 56°C for 60 s, followed by melting curve analysis. The analyzed miRNAs primer information can be found in Table S6. qPCR amplification curves were evaluated with CFX Manager software (Bio-Rad). The specificity of the amplification was confirmed by the melting curve analysis. Then, the expression level of each miRNA was calculated using the 2^−ΔCq^ method (where ΔCq = CqmiRNA − Cq hsa-miR-103a-3p). The normalized miRNA levels were further log2 converted.

### Functional enrichment analysis

Functional enrichment of predicted genes of differentially expressed miRNAs was analyzed using FunRich software (http://www.funrich.org/). The KEGG, GO, Uniprot, Reactome, and FunRich databases were used to identify the molecular functions and biological processes. Finally, the miRNA TissueAtlas (https://ccb-web.cs.uni-saarland.de/tissueatlas) was used to determine the expression of the selected miRNA within the tissues.

### Statistical analysis

All statistical analyses were performed using GraphPad Prism 8 software. Comparisons among multiple groups were performed using one-way analysis of variance, followed by non-parametric Kruskal-Wallis rank tests. ROC curves were applied to characterize the diagnostic performance of both each and combined miRNAs. ROC curves were generated by plotting sensitivity against 100% specificity, indicating the AUC and 95% confidence intervals. Pearson correlation coefficient was used for correlations between log2 miRNAs versus clinical parameters in critical COVID-19 patients. Differences were considered statistically significant at p < 0.05.

## Supplemental information

Document S1. Figures S1–S3 and Tables S1–S3, S5, and S6

Table S4. Functional enrichment analysis based on hsa-miR-32-5p, hsa-miR-98-3p, hsa-miR-423-3p, and hsa-miR-1246 target genes

Document S3. Article plus supplemental information
