# Supplementary material for: Serum microRNAs targeting ACE2 and RAB14 genes distinguish asymptomatic from critical COVID-19 patients
Source: Mol Ther Nucleic Acids. 2022 Jun 11;29:76–87. doi: 10.1016/j.omtn.2022.06.006 (PMC9188110; doi:10.1016/j.omtn.2022.06.006)
Supplement: Document S1. Figures S1–S3 and Tables S1–S3, S5, and S6 [file mmc1.pdf]

OMTN, Volume *29*

Supplemental information

Serum microRNAs targeting *ACE2* and *RAB14* genes distinguish asymptomatic from critical COVID-19 patients

Maria Calderon-Dominguez, Eva Trejo-Gutierrez, Almudena González-Rovira, Lucía Beltrán-Camacho, Marta Rojas-Torres, Sara Eslava-Alcón, Daniel Sanchez-Morillo, Juan Calderon-Dominguez, Ma Pilar Martinez-Nicolás, Estibaliz Gonzalez-Beitia, Ma Dolores Nieto-Martín, Teresa Trujillo-Soto, Manuel A. Rodríguez-Iglesias, Juan A. Moreno, Rafael Moreno-Luna, and Ma Carmen Durán-Ruiz

Table S1. Bibliographic references of miRNAs related to the respiratory system

miRNAs	DOI or PMID
miR-32-5p	10.1517/17530050902852697<br>10.7150/jca.48387<br>10.1186/s12864-018-5416-0<br>10.1165/rcmb.2020-0112OC<br>10.3892/mmr.2021.11851<br>10.3389/fped.2021.671107
miR-98-3p	10.1177/1010428317691659<br>10.1002/CAM4.3379<br>10.3389/fmolb.2021.640042<br>10.1002/jcb.29612
miR-214-3p	10.1161/CIRCRESAHA.119.315067<br>10.1164/rccm.201807-1237OC<br>10.12659/MSM.915709<br>10.1038/s41389-019-0151-1<br>10.1089/ars.2019.7965
miR-421	10.1159/000503020<br>10.3892/ol.2020.12169<br>10.2147/CMAR.S167432<br>10.26355/eurrev_201905_17805<br>10.2147/CMAR.S258887<br>10.1016/j.prp.2019.152555<br>10.1042/CS20130420<br>10.1158/1055-9965.EPI-15-0161<br>10.1002/jcb.28945<br>10.2147/CMAR.S248869
miR-423-3p	10.1016/j.tube.2014.10.011<br>10.1186/s12920-020-00748-3<br>10.14336/AD.2015.0620<br>10.1186/s13000-019-0831-3<br>10.1016/j.lungcan.2017.10.002<br>10.1186/s11658-021-00247-y<br>10.3390/ijms21155381
miR-1246	10.1080/07391102.2020.1833760<br>10.4143/crt.2018.638<br>10.1164/rccm.202003-0541OC<br>10.7717/peerj.9943<br>10.1016/j.lungcan.2015.11.013<br>10.2147/JAA.S331090<br>PMID: 28386354

Table S2. The binding site position of candidate miRNAs at 3'UTR mRNA

3'UTR mRNA	miRNA name	Number of binding sites	Position in 3´UTR sequence (canonical matched sites)
ACE2	hsa-miR-421	3	2567-2591, 2567-2587, 2488-2509
	hsa-miR-1246	3	1285-1317, 450-468, 2397-2433
TMPRSS2	hsa-miR-214-3p	5	1880-1905, 1942-1962, 1770-1798, 2422-2438, 2340-2355
RAB14	hsa-miR-32-5p	2	2822-2843, 1853-1870
	hsa-miR-98-3p	6	1527-1543, 3087-3093, 1578-1583, 1941-1947, 2500-2537, 2237-2269
	hsa-miR-214-3p	6	1824-1845, 2419-2439, 2027-2056, 2719-2744, 2877-2914, 1855-1890
	hsa-miR-423-3p	1	1161-1175
	hsa-miR-1246	3	2666-2692, 3382-3407, 1423-1456

Table S3. Clinical variables of the study population

	COVID-19 negative control N=16	Asymptomatic IgG+ COVID-19 patients N=16	Critical COVID-19 patients N=17
Age (years)	50±2.18	49.31±1.99	44.88±4.35
Sex (male)	6.3 %	31.3 %	35.5 %
Symptom (%)	12.2 %	50 %	100 %
Positive qRT-PCR.COVID-19 (%)	0 %	0 %	100 %
Positive IgG	0 %	100 %	0 %
Risk factor	12.5 %	18.8 %	52.92 %
Obesity	0 %	12.5 %	23.52 %
Dyslipidemia	6.3 %	0 %	11.75 %
Type 2 Diabetes	0 %	0 %	5.88 %
Asthma	6.3 %	0 %	5.9 %
Arterial hypertension	6.3 %	6.3 %	23.52 %
Smoker	25 %	18.75 %	5.88 %

All values are expressed as mean ± SEM

Table S4. Functional enrichment analysis based on based on hsa-miR-32-5p, hsa-miR-98-3p, hsa-miR-423-3p and hsa-miR-1246 target genes. This Table is included as an excel file.

Table S5. miRNA expression data in COVID-19 samples compared to other respiratory disorders.

miRNAs	Expression in our Critical COVID-19 patients	Expression in other respiratory disorders	Sample	DOI or PMID
**hsa-miR-32-5p**	Upregulated	Downregulated (*p*=0.01) (Figure R2.Q2.B)	NSCLC tissues	10.7150/jca.48387
		Downregulated (*p*<0.05) (Figure R2.Q2.A)	Plasma from ARDS patients	10.1165/rcmb.2020-0112OC
		Upregulated (FC=4.9954; *p*=0.003)	Peripheral blood from BPD patients	10.1155/2018/6204128
		Downregulated (FC= 8.09; *p*= 0.006) Data available in the Supplementary information	Lung tissues from CPAMs patients	10.3389/fped.2021.671107
**miR-98-3p**	Upregulated	Upregulated (FC=3.8078613; *p*=0.009)	NSCLC cell line	10.1177/1010428317691659
		Downregulated (FC=0.12; *p*=0.022)	Plasma from LAC patients	10.1002/jcb.29612
		Upregulated (FC= 20.64; *p*=0.0091)	Plasma from ARDS patients	10.3389/fmolb.2021.640042
**hsa-miR-423-3p**	Upregulated	Upregulated (FC= 5.95; *p*<0.05)	Serum from LC patients	10.1016/j.lungcan.2017.10.002
**hsa-miR-1246**	Upregulated	Downregulated (FC= 1.46; *p*=0.027)	Serum from COPD patients	10.2147/COPD.S271864
		Upregulated (*p*<0.05) (Figure R2.Q2.C and D)	NSCLC tissues and LUAD tissues	10.1038/ncomms11702

Abbreviations: **ARDS,** acute respiratory distress syndrome; **BPD**, bronchopulmonary dysplasia; **COPD,** chronic obstructive pulmonary disease; **CPAMs**, congenital pulmonary airway malformations; **FC**, fold change; **LAC**, lung adenocarcinoma; **LC**, lung cancer**; LUAD**, lung squamous carcinoma; **NSCLC**, Non-small cell lung cancer

Table S6. Analyzed miRNAs and their manufacturer's information

miRNA ID	Product Name	GeneGlobe Id	Catalog Number	Accession number	Sequence
hsa-miR-32-5p	hsa-miR-32-5p miRCURY LNA miRNA PCR Assay	YP00204792	339306	MIMAT0000090	5'UAUUGCACAUUACUAAGUUGCA
hsa-miR-98-3p	hsa-miR-98-3p miRCURY LNA miRNA PCR	YP02106353	339306	MIMAT0022842	5'CUAUACAACUUACUACUUUCCC
hsa-miR-214-3p	hsa-miR-214-3p miRCURY LNA miRNA PCR Assay	YP00204510	339306	MIMAT0000271	5'ACAGCAGGCACAGACAGGCAGU
hsa-miR-421	hsa-miR-421 miRCURY LNA miRNA PCR Assay	YP00204603	339306	MIMAT0003339	5'AUCAACAGACAUUAAUUGGGCGC
hsa-miR-423-3p	hsa-miR-423-3p miRCURY LNA miRNA PCR Assay	YP00204488	339306	MIMAT0001340	5'AGCUCGGUCUGAGGCCCCUCAGU
hsa-miR-1246	hsa-miR-1246 miRCURY LNA miRNA PCR Assay	YP00205630	339306	MIMAT0005898	5'AAUGGAUUUUUGGAGCAGG
hsa-miR-103a-3p	hsa-miR-103a-3p miRCURY LNA miRNA PCR Assay	YP00204063	339306	MIMAT0000101	5'AGCAGCAUUGUACAGGGCUAUGA

Figure S1

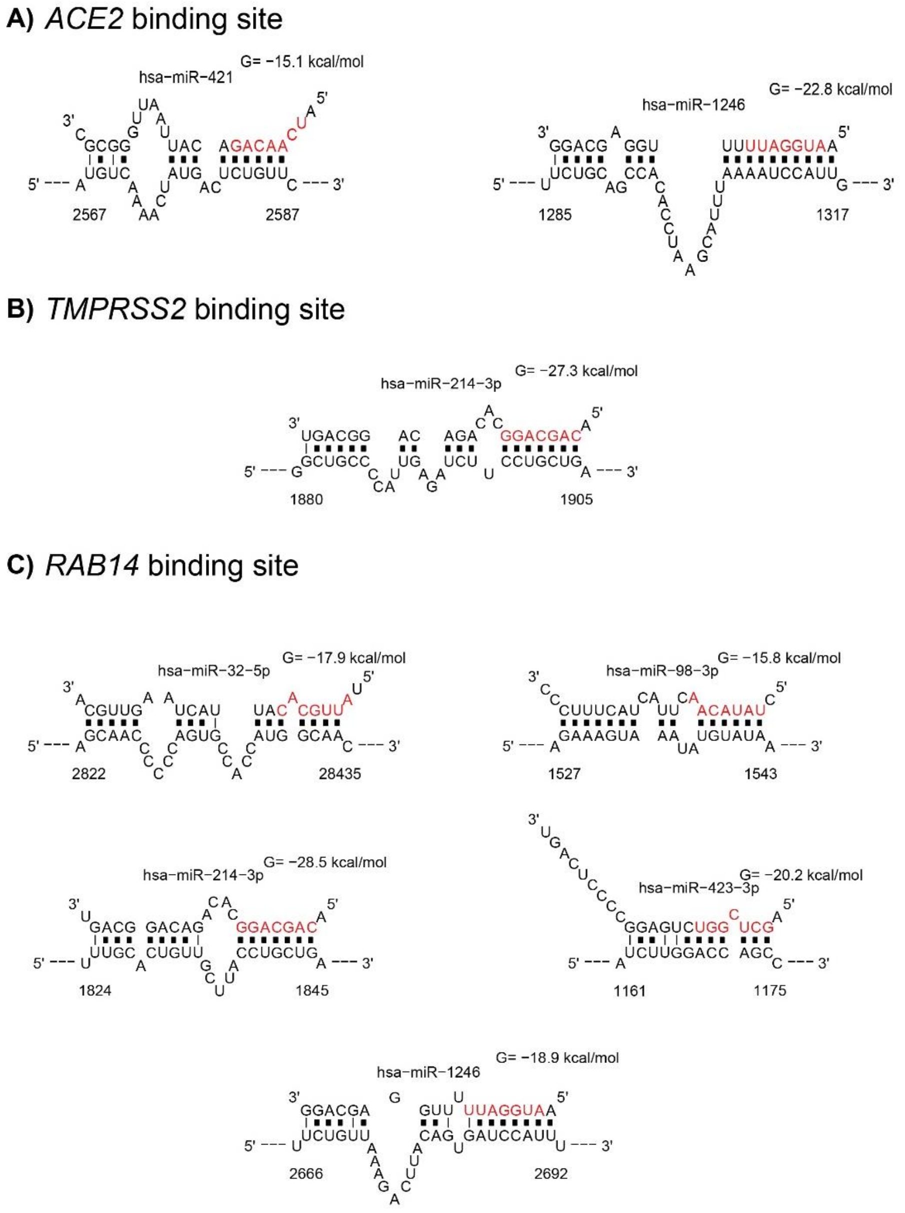

Figure S1. Predicted binding site of selected miRNAs in the ACE2, TMPRSS2, and RAB14 3′-UTR. The figure summarizes the information provided by STarMir and RNA hybrid on location, sequence complementarity, free energy, and target site accessibility. ACE2, TMPRSS2, and RAB14 3′-UTR sequences are written in black, whereas the selected microRNA seed regions appear in red. A) Binding site of hsa-miR-421 and hsa -miR-1246 in 3'UTR of ACE2 mRNA. B) Binding site of hsa-miR-214-3p in 3'UTR of TMPRSS2. C) Binding site of hsa-miR-32-5p, hsa-miR-98-3p, hsa-miR-214-3p, hsa-miR-423-3p, and hsa-miR-1246 in 3'UTR of ACE2 mRNA.

Figure S2

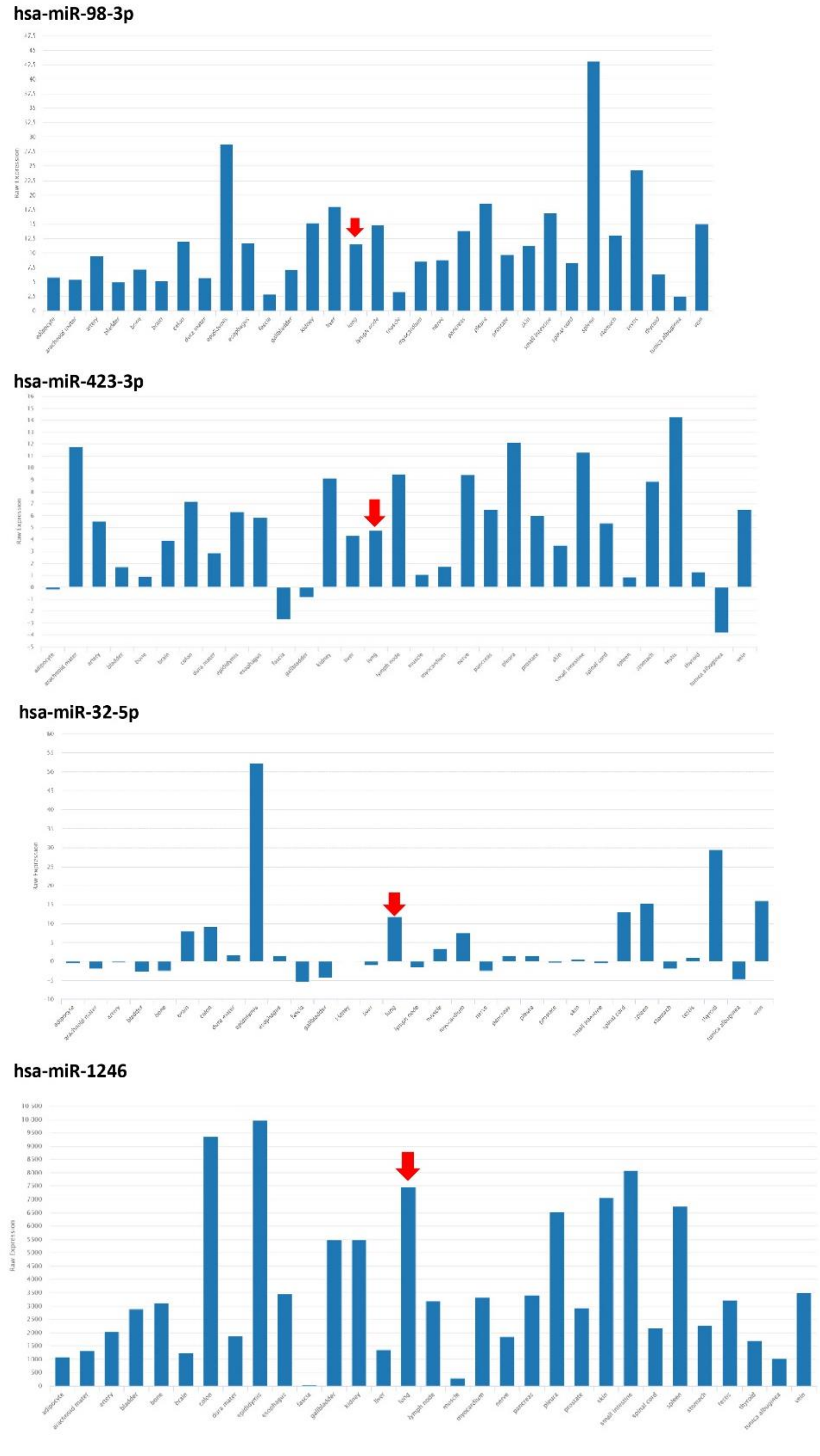

Figure S2. miRNAs normalized expression across organ systems. The figure summarizes the information provided by the Human miRNA Tissue atlas (https://ccb-web.cs.uni-saarland.de/tissueatlas). The red flag indicated the predicted expression of hsa-miR-98-3p, hsa-miR-32-5p, hsa-miR-423-3p, and hsa-miR-1246 in the lungs.

Figure S3

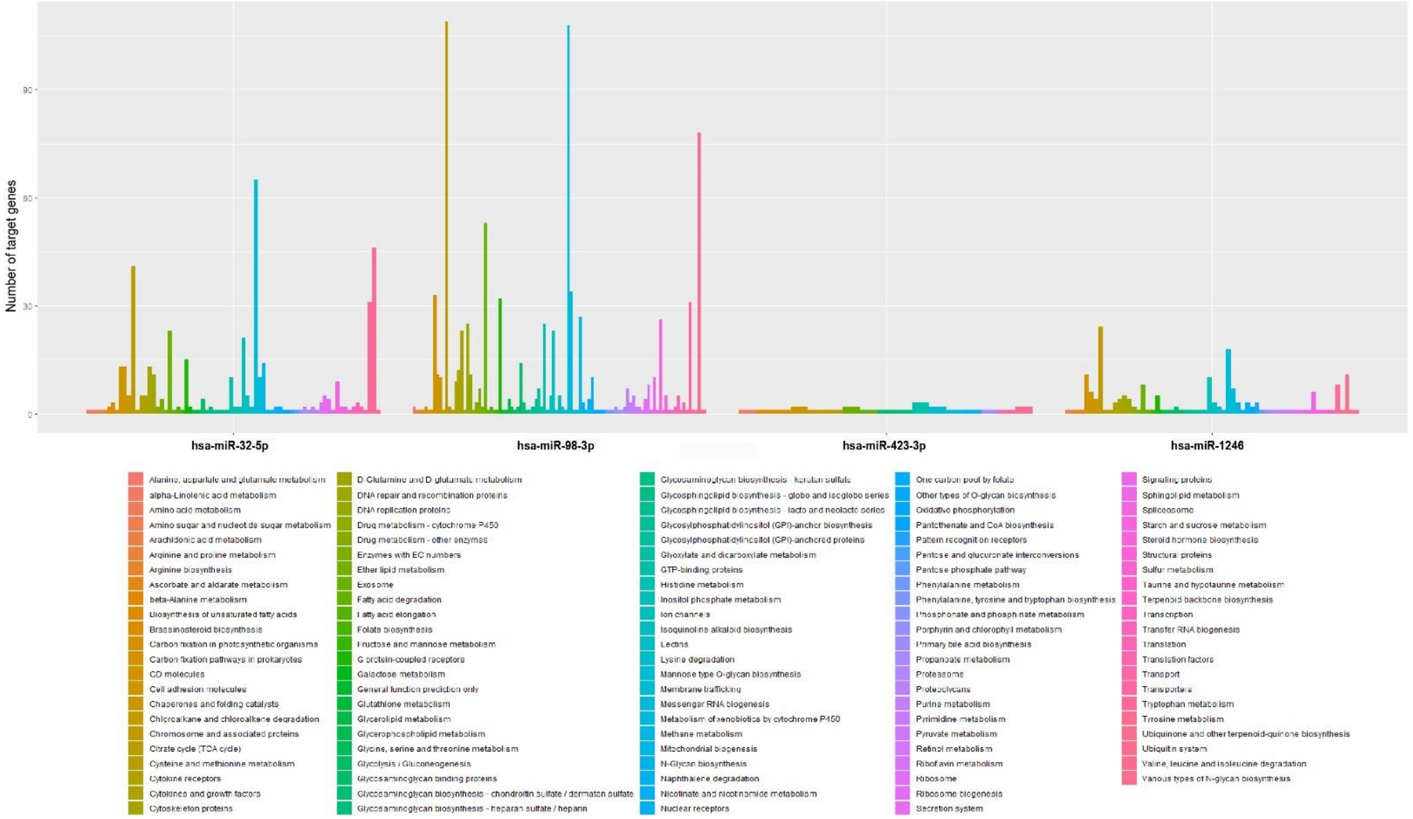

Figure S3. Extended KEGG categorization of targeted genes of hsa-miR-32-5p, hsa-miR-98-3p, hsa-miR-423-3p, and hsa-miR-1246. The x-axis indicates KEGG categories, and the y-axis indicates the number of gene targets.
