# Supplementary material for: Serum microRNAs targeting ACE2 and RAB14 genes distinguish asymptomatic from critical COVID-19 patients
Source: Mol Ther Nucleic Acids. 2022 Jun 11;29:76–87. doi: 10.1016/j.omtn.2022.06.006 (PMC9188110; doi:10.1016/j.omtn.2022.06.006)
Supplement: Document S3. Article plus supplemental information [file mmc3.pdf]

Molecular Therapy
Nucleic Acids
Original Article

Serum microRNAs targeting *ACE2* and *RAB14* genes distinguish asymptomatic from critical COVID-19 patients

Maria Calderon-Dominguez,[1,2] Eva Trejo-Gutierrez,[1] Almudena González-Rovira,[1,2] Lucía Beltrán-Camacho,[1,2] Marta Rojas-Torres,[1,2] Sara Eslava-Alcón,[1,2] Daniel Sanchez-Morillo,[2,3] Juan Calderon-Dominguez,[2] Mª Pilar Martinez-Nicolás,[4] Estibaliz Gonzalez-Beitia,[4] Mª Dolores Nieto-Martín,[5] Teresa Trujillo-Soto,[2,6] Manuel A. Rodríguez-Iglesias,[1,2,6] Juan A. Moreno,[7,8] Rafael Moreno-Luna,[9,10] and Mª Carmen Durán-Ruiz[1,2,10]

[1]Biomedicine, Biotechnology and Public Health Department, Cádiz University, 11002 Cádiz, Spain; [2]Biomedical Research and Innovation Institute of Cadiz (INiBICA), 11009 Cádiz, Spain; [3]Biomedical Engineering and Telemedicine Research Group, Department of Automation Engineering, Electronics and Computer Architecture and Networks, Universidad de Cádiz, 11009 Cádiz, Spain; [4]Occupational Health Service, National Paraplegic Hospital, SESCAM, 45071 Toledo, Spain; [5]Internal Medicine Department, University Hospital Virgen del Rocío, Seville, Spain; [6]UGC Microbiología, University Hospital Puerta del Mar, Avda. Ana de Viya 21, 11009 Cádiz, Spain; [7]Cell Biology, Physiology and Immunology Department, Agrifood Campus of International Excellence (ceiA3), University of Cordoba, 14014 Córdoba, Spain; [8]Maimonides Biomedical Research Institute of Cordoba (IMIBIC), UGC Nephrology, Hospital Universitario Reina Sofia, 14004 Cordoba, Spain; [9]Laboratory of Neuroinflammation, National Paraplegic Hospital, SESCAM, 45071 Toledo, Spain

Despite the extraordinary advances achieved to beat COVID-19 disease, many questions remain unsolved, including the mechanisms of action of SARS-CoV-2 and which factors determine why individuals respond so differently to the viral infection. Herein, we performed an *in silico* analysis to identify host microRNA targeting *ACE2*, *TMPRSS2*, and/or RAB14, all genes known to participate in viral entry and replication. Next, the levels of six microRNA candidates previously linked to viral and respiratory-related pathologies were measured in the serum of COVID-19-negative controls (n = 16), IgG-positive COVID-19 asymptomatic individuals (n = 16), and critical COVID-19 patients (n = 17). Four of the peripheral microRNAs analyzed (hsa-miR-32-5p, hsa-miR-98-3p, hsa-miR-423-3p, and hsa-miR-1246) were upregulated in COVID-19 critical patients compared with COVID-19-negative controls. Moreover, hsa-miR-32-5p and hsa-miR-1246 levels were also altered in critical versus asymptomatic individuals. Furthermore, these microRNA target genes were related to viral infection, inflammatory response, and coagulation-related processes. In conclusion, SARS-CoV-2 promotes the alteration of microRNAs targeting the expression of key proteins for viral entry and replication, and these changes are associated with disease severity. The microRNAs identified could be taken as potential biomarkers of COVID-19 progression as well as candidates for future therapeutic approaches against this disease.

INTRODUCTION

On 30 January, 2020, the World Health Organization declared coronavirus disease 19 (COVID-19) a public health emergency of international concern, becoming a global pandemic on 11 March, 2020.[1] COVID-19 has drastically affected the entire world, at both economic and public health levels, with millions of deaths worldwide.[2,3] The enormous effort in the development of vaccines against the severe acute respiratory syndrome coronavirus 2 (SARS-CoV-2) has significantly decreased the rhythm of disease progression. Unfortunately, it is still not possible to predict the efficacy and durability of immunity after vaccination, mainly taking into account the viral mutations constantly arising, as in the case of Omicron, one of the last variants identified.[4,5] Thus, major efforts are actually focused on understanding the molecular mechanisms of SARS-CoV-2 infection, as well as on the identification of therapeutic targets against COVID-19 and potential biomarkers to prevent its progression.[6] Two clear candidates in this therapeutic approach are angiotensin-converting enzyme 2 (ACE2), one of the main co-factors required by SARS-CoV-2 to access human host cells, and the transmembrane protease serine 2 (TMPRSS2), which is responsible for priming of the viral spike protein, a step required to allow the virus-host cell membrane fusion and further internalization of the virus.[7,8] ACE2 and TMPRSS2 have received more attention; however, other host-cell proteins related to SARS-CoV-2 replication are also playing an essential role during viral infection, promoting its survival. For instance, the interaction of SARS-CoV-2 with host proteins involved in the assembly and viral trafficking, such as Ras-related protein Rab-14 (RAB14), may favor SARS-CoV-2 replication.[9,10] Hence, the use of agents

Received 9 March 2022; accepted 5 June 2022;
https://doi.org/10.1016/j.omtn.2022.06.006.

[10]These authors contributed equally

Correspondence: Mª Carmen Duran-Ruiz, PhD, Biomedicine, Biotechnology and Public Health Department, Science Faculty, Institute of Biomedical Research of Cádiz (INiBICA), Cádiz University, Torre Sur. Avda. República Saharaui S/N, Polígono Río San Pedro, C.P. 11519 Puerto Real, Spain.
E-mail: maricarmen.duran@gm.uca.es

 © 2022 The Authors.
This is an open access article under the CC BY license (http://creativecommons.org/licenses/by/4.0/).

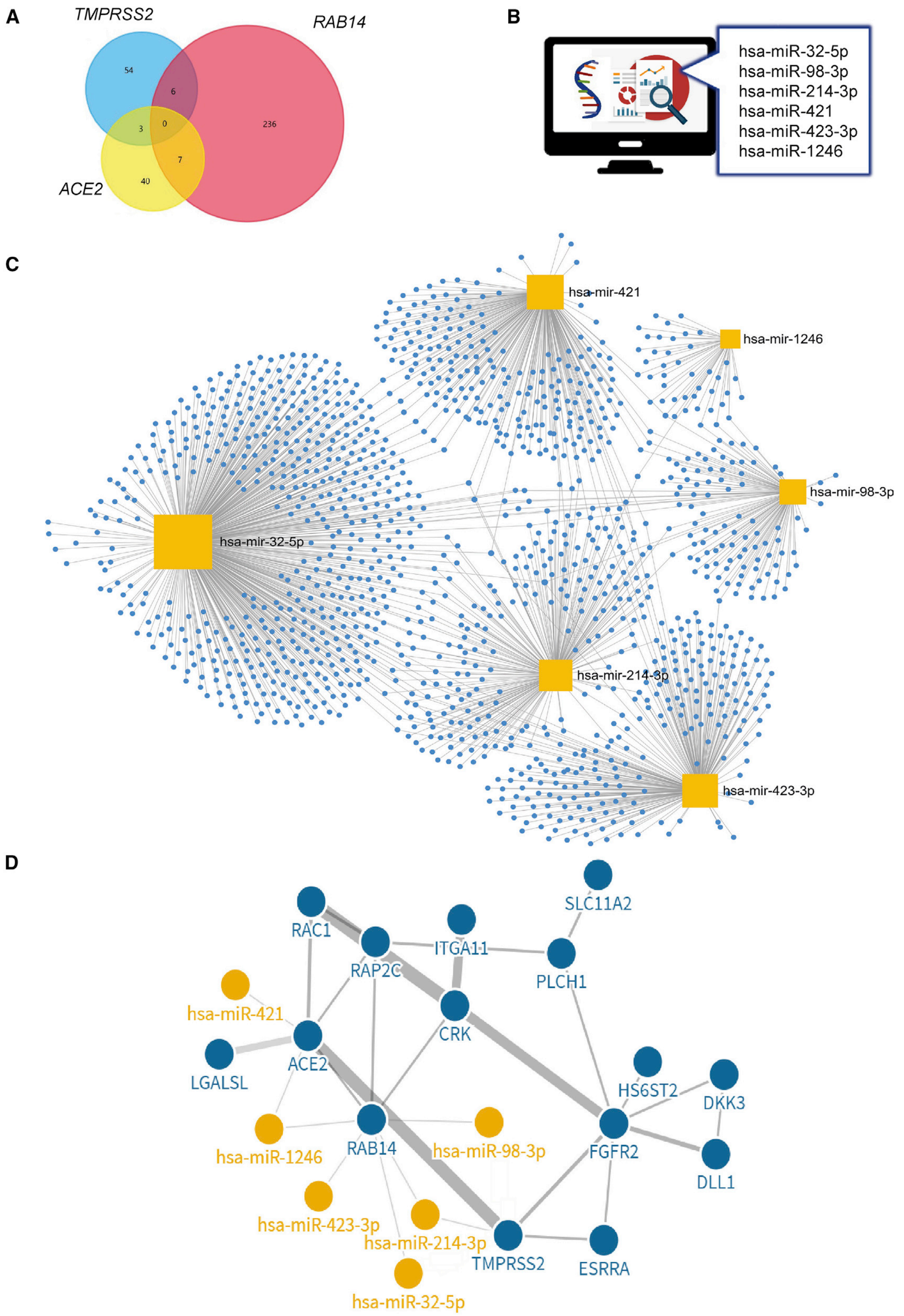

(legend on next page)

blocking or interfering with the interaction between SARS-CoV-2 and ACE2, TMPRSS2, or RAB14 might help to prevent or reduce the virus entry and replication. Indeed, some inhibitors are being tested. That is the case of camostat mesylate, targeting TMPRSS2, which seems to block SARS-CoV-2 infection of lung cells.[8] Alternatively, the use of inhibitors against SARS-CoV-2 mRNA translation are also of interest.[11,12] In this regard, the application of specific microRNAs (miRNAs) to regulate the expression of essential proteins for the SARS-CoV-2 infection process constitutes a promising but also unexplored approach.[13,14]

MiRNAs are small (18–22 nucleotides) highly conserved, non-coding single-stranded ribonucleic acids (RNAs), which appear to be involved in many physiological processes as well as in different diseases, participating, among others, in the modulation of viral infection and host defense.[15] To date, several miRNAs have been found in altered levels in the blood of individuals after viral infection, including COVID-19 patients.[6,16,17] Thus, miRNAs not only constitute promising therapeutic tools[17] but they are also considered as potential prognostic markers for SARS-CoV-2 infection.[18]

In this study, based on the literature and *in silico* results, we focus on analyzing the levels of miRNA targeting *ACE2*, *TMPRSS2*, and *RAB14*, all associated with viral entry and replication in the serum of COVID-19 patients. Our results indicate that peripheral hsa-miR-32-5p, hsa-miR-98-3p, hsa-miR-423-3p, and has-miR-1246 increased in critical COVID-19 patients. The functional pathways in which these molecules participate, as well as the potential use of these molecules as biomarkers, are discussed.

RESULTS

In silico analyses to predict miRNAs targeting *ACE2*, *TMPRSS2*, and *RAB14*

The miRNAs potentially targeting *ACE2*, *TMPRSS2*, and/or *RAB14* genes were determined by *in silico* analysis using different miRNA target prediction tools (Figure 1A). Among all miRNA predicted to target these genes, only a few were related to the respiratory system or described as post-transcriptional regulators (Table S1). Finally, six miRNAs (hsa-miR-32-5p, hsa-miR-98-3p, hsa-miR-214-3p, hsa-miR-421, hsa-miR-423-3p, and hsa-miR-1246) were selected as our targets of interest (Figure 1B).

Next, the number of binding sites of all six miRNAs were analyzed, indicating the number of all selected miRNAs in the 3′ UTR of *ACE2*, *TMPRSS2*, and *RAB14* (Table S2). As previously described,[19] a match of six nucleotides between miRNA and 3′ UTR sequence may be considered as a potential seeding region (Figure S1). hsa-miR-98-3p and hsa-miR-423-3p displayed the highest number, with six 3′ UTR binding sites for the *RAB14* gene.

Furthermore, the target genes of these six miRNA candidates were also predicted (Figure 1C). According to protein-protein interaction networks analysis of the shared target genes (Figure 1D), they all displayed a narrow interaction with each other ($p = 0.003$).

Identification of differentially expressed miRNAs in critical COVID-19 patients

Once the six miRNAs candidates were selected, the serum levels were analyzed by qPCR in our study population. A graphical representation of some characteristics registered for the study population is shown in Figure 2 (extended in Table S3). Most of the patients enrolled were female (Figure 2A). The mean ages for COVID-19-negative controls, asymptomatic IgG-positive subjects, and critical COVID-19 patients were 50 ± 2.18, 49.31 ± 1.99, and 44.88 ± 4.35 years, respectively (Figure 2B). Critical patients presented some risk factors, such as obesity (23.5%), arterial hypertension (AHT) (24%), diabetes mellitus type 2 (T2D) (5.88%), asthma (5.9%), and thalassemia (5.9%) (Figure 2C). Finally, the percentage of smokers in critical COVID-19 patients was 5.8%, 25% in COVID-19-negative controls, and 18.75% in asymptomatic individuals. A schematic representation of the study workflow is shown in Figure 2D.

The miRNA levels detected in the serum samples are shown in Table 1. A total of four peripheral miRNAs (hsa-miR-32-5p, hsa-miR-98-3p, hsa-miR-423-3p, and hsa-miR-1246) were differentially expressed in our population (Figure 3). Thus, hsa-miR-98-3p, hsa-miR-423-3p, and hsa-miR-1246 were significantly increased in critical patients compared with COVID-19-negative subjects (Figures 3B–3D). Moreover, peripheral hsa-miR-32-5p and hsa-miR-1246 were differentially expressed in critical COVID-19 patients compared with asymptomatic IgG-positive donors (Figures 3A and 3D). In addition, according to the miRNA TissueAtlas platform, all four miRNAs have been found expressed in lungs, among other tissues (Figure S2).

Diagnostic potential of serum miRNAs and the association with critical COVID-19 patients

Next, to analyze the diagnostic value, the area under the curve-receiver operating characteristic (AUC-ROC) was compared for the single and combined differentially expressed miRNAs (Table 2). Although the combined miRNA AUC values were significant, none of them reached a value higher than single miRNAs. The ROC curve of single hsa-miR-32-5p, hsa-miR-98-3p, hsa-miR-423-3p, and hsa-miR-1246 revealed the probability to use them as valuable biomarkers to diagnose critical COVID-19 patients from COVID-19-negative controls and asymptomatic IgG-positive individuals (Figure 4A-D). The highest discriminatory power achieved by a single miRNA was acquired for hsa-miR-1246, with an AUC of 0.875 (95% CI: 0.755–0.995; $p = 0.0002$) (Figure 4D).

Figure 1. Bioinformatic analysis of the *ACE2*, *TMPRSS2*, and *RAB14* genes to predict their potential miRNAs

(A) Venn diagram of the predicted miRNAs for *ACE2*, *TMPRSS*, and *RAB14* genes. (B) miRNAs related to the respiratory system were selected from bibliographic search. (C) The miRNA-mRNA network of selected miRNAs. (D) Interaction network of the shared target genes (blue) and the selected miRNAs (yellow). The size of the gray lines represents the degree of interaction between proteins.

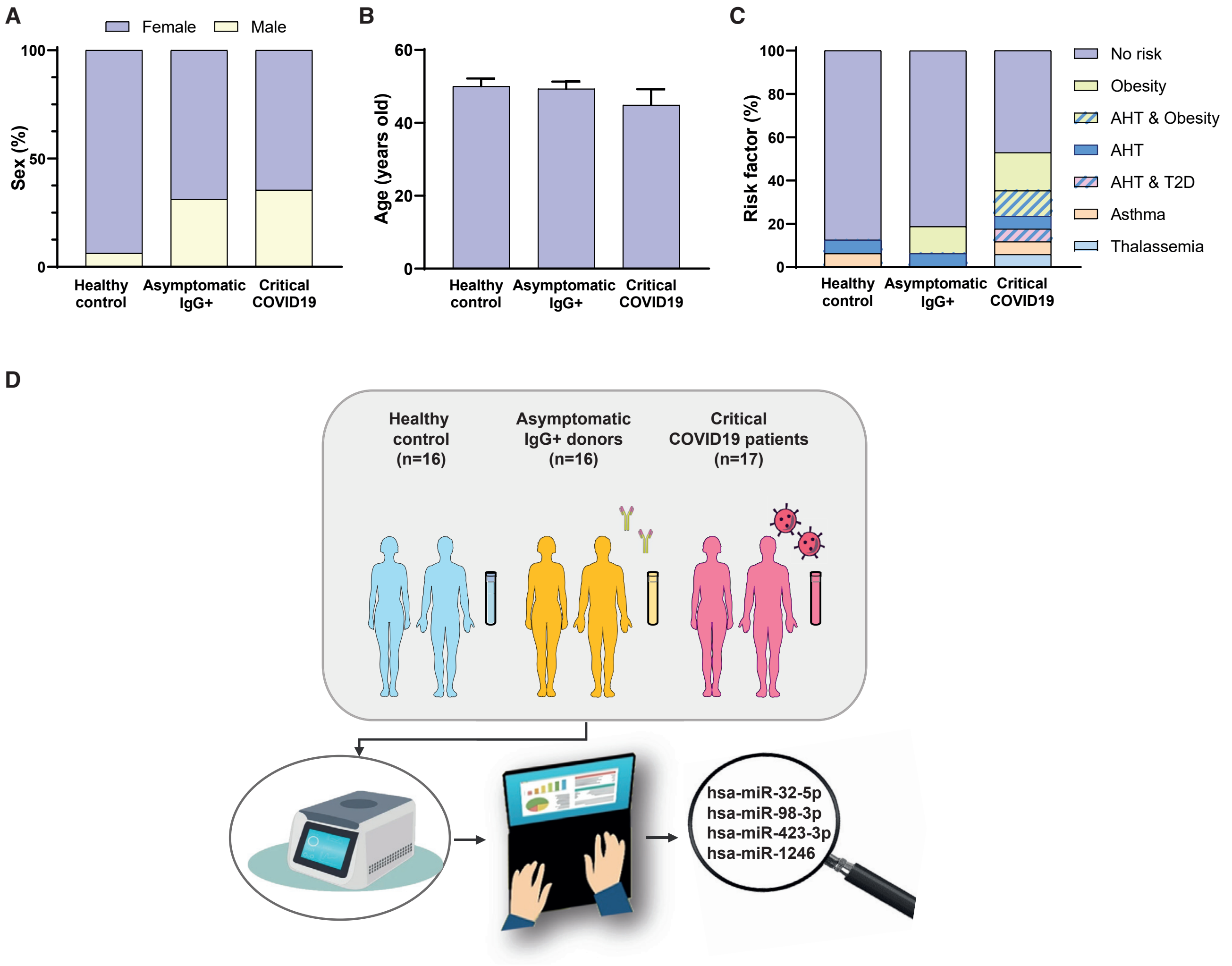

Figure 2. Study population and flow diagram of the experimental progress.

Clinical variables of the study population: (A) Gender (%); (B) Age, represented as the mean +- SEM; (C) Risk factors found for individuals in each group (healthy controls, asymptomatic IgG+ individuals and critical COVID19 patients). (D) Flowchart of the study population and the protocol of the experimental steps, including qPCR analysis and the selected serum miRNAs.

Finally, the association between peripheral miRNA levels and clinical variables was also evaluated (Table 3). The circulating hsa-miR-32-5p and hsa-miR1246 levels showed a negative correlation with the presence of risk factor. Furthermore, the circulating hsa-miR-32-5p levels indicated a positive correlation with the age and severity of COVID-19, which could be possible since miRNA expression is related to age.[20] On the other hand, although a differential expression of miRNAs has been described between males and females, only the levels of hsa-98-3p showed an association with the gender.[21]

Functional enrichment analysis

A total of 2,812 genes were detected as potential targets of our four differentially quantified serum miRNAs. Remarkably, *RAB14* was the only common target gene for hsa-miR-32-5p, hsa-miR-98-3p, hsa-miR-423-3p, and hsa-miR-1246 (Figure 5A). According to the functional analysis performed via the KEGG database (Figure 5B), the genes targeted by these four miRNAs were mainly involved in the processing of genetic information, cell signaling, and other cell-related processes (extended in Figure S3).

Moreover, the targeted genes were involved in several biological processes (Figures 6A–6E), which were all significantly enriched (extended in Table S4). Within the cellular process category, many genes correlated with vesicle trafficking and cell-cell interactions, as well as regulation of cell cycle, cell proliferation, migration, or even regulation of the NLRP3 inflammasome complex (Figure 6A). Many genes were involved in RNA transcription and mRNA processing and transport, which correlates with the involvement of miRNAs in the regulation of RNA polymerase I and II expression, mRNA processing, stability, and splicing, among others (Figure 6B). Moreover,

Table 1. Peripheral miRNAs levels in the study groups

	COVID-19-negative control			Asymptomatic IgG-positive COVID-19			Critical COVID-19 patients					
	Median	Q1	Q3	Median	Q1	Q3	p value versus COVID-19-negative controls	Median	Q1	Q3	p value versus COVID-19-negative controls	p value versus aymptomatic IgG positive patients
hsa-miR-32-5p	5.359	5.161	5.444	5.186	4.677	5.324	0.1616	5.356	5.191	5.789	>0.9999	**0.0330**
hsa-miR-98-3p	3.201	2.905	3.362	3.377	3.087	3.624	0.2258	3.469	3.269	3.815	**0.0254**	>0.9999
hsa-miR-214-3p	3.869	3.737	4.125	3.793	3.534	4.164	>0.999	3.756	3.525	3.926	0.8096	>0.9999
hsa-miR-421	4.037	3.919	4.137	4.113	4.059	4.257	0.4034	4.148	4.074	4.361	0.1692	>0.9999
hsa-miR-423-3p	4.961	4.842	5.048	5.094	5.037	5.181	0.1207	5.192	4.969	5.289	**0.0048**	0.7860
hsa-miR-1246	5.343	5.155	5.583	5.361	5.121	5.605	>0.9999	6.009	5.695	6.846	**0.0007**	**0.0005**

Data presented as median (Q1-Q3). Coefficient significant at $p < 0.05$ (highlighed in bold).

these genes were also directly linked to the viral process, including viral RNA replication and viral entry into the host nucleus or regulation of the viral genome replication (Figure 6D). Also, all four miRNAs have been previously associated to respiratory-related diseases (extended in Table S5). Finally, several genes were associated to blood-related processes, such as coagulation, vessel development, morphogenesis, and remodeling (Figure 6C), and also to neuroepithelial cell differentiation and regulation of neural precursor cell proliferation, among others (Figures 6D and 6E).

DISCUSSION

After 2 years since the first person was diagnosed with COVID-19, more than 447 million cases have been reported around the world, including more than 6 million deaths.[22] To date, numerous diagnostic methods and vaccines have been developed in record time, thanks to extraordinary and unprecedented scientific and clinical efforts. Nevertheless, the mechanisms of action of SARS-CoV-2, as well as the potential secondary effects that this virus exerts over the organism are not fully understood yet.

One of the unanswered questions regarding COVID-19 is why some people infected with SARS-CoV-2 present severe symptoms, even though others do not. Most patients show mild to moderate symptoms such as fever, persistent dry cough, body aches, and occasional dyspnea. However, a small fraction of patients may also present acute respiratory failure and acute respiratory distress syndrome associated with multiple organ failure.[23,24] Consequently, knowing an individual's susceptibility to SARS-CoV-2 infection, through identifying critical biomarkers, may guide future treatment strategies for the disease and its different phases. In this sense, peripheral miRNAs have been remarked as potential biomarkers for COVID-19 treatment and diagnosis since they can play an essential role in the pathogenesis of its infection.[6,25]

In this study, we focused on the identification of miRNAs targeting *ACE2*, *TMPRSS2*, and *RAB14* genes, due to their direct or indirect association with SARS-CoV-2 infection. While other miRNAs had been recently associated with the pathology of COVID-19, being upregulated in acute and critical patients compared with controls (i.e., miR-29a-3p, -146a-3p, -155-5p,[25] or miR-6501-5p and miR-618[6]), none of them targeted *ACE2*, *TMPRSS2*, or *RAB14*. Notably, four of the six miRNAs selected from the *in silico* analysis (hsa-miR-32-5p, hsa-miR-98-3p, hsa-miR-423-3p, and hsa-miR-1246) were significantly increased in the serum of critical COVID-19 patients. In addition, hsa-miR1246 could discriminate between IgG-positive asymptomatic subjects and critical COVID-19 patients. Furthermore, based on their AUC-ROC values, these four miRNAs could be considered novel biomarkers with high-yield diagnostic accuracy. In addition, hsa-miR-32-5p and hsa-miR-1246 correlated with the presence of obesity,[26,27] AHT,[28] T2D,[29] asthma,[30] or thalassemia,[31] which have been described as risk factors for critical COVID-19 patients.

Remarkably, none of the four miRNAs found altered in the serum of critical COVID-19 patients had *TMPRSS2* as a target gene, while they all targeted *RAB14*. Among them, hsa-miR-32-5p has been associated *in silico* to *RAB14* in pancreatic and colon tumors.[32] Moreover, hsa-miR-32-5p and miR-98-3p, also targeting *RAB14*, were identified as crucial lung cancer-associated miRNAs,[33–35] and their deregulation levels were identified in several respiratory disorders, such as acute respiratory distress syndrome,[36,37] bronchopulmonary dysplasia,[38] and congenital pulmonary airway malformations.[39] Besides, these miRNAs have been previously related with viral processes. For instance, miR-32-5p has a significant regulatory role in avian hepatitis A viral infection,[40] visna maedi virus,[41] and caprine arthritis encephalitis viruses,[41] while the deregulation of the expression miR-98 has been described in a mouse model of West Nile virus neuropathogenesis.[42]

Apart from these two miRNAs, hsa-miR-423-3p also exhibited *RAB14* as a unique target gene. While previous studies found increased levels of the circulating 5′ form (hsa-miR-423-5p) in COVID-19 patients, nothing was described concerning hsa-miR-423-3p.[43] This miRNA has been defined as a biomarker of lung cancer,[44] and it has also been related to tuberculosis and endocytosis pathways, in which the RAB protein family plays a crucial role.[45]

Finally, according to our *in silico* analysis, hsa-miR-1246 was the only miRNA that targeted both *ACE2* and *RAB14* genes. As mentioned

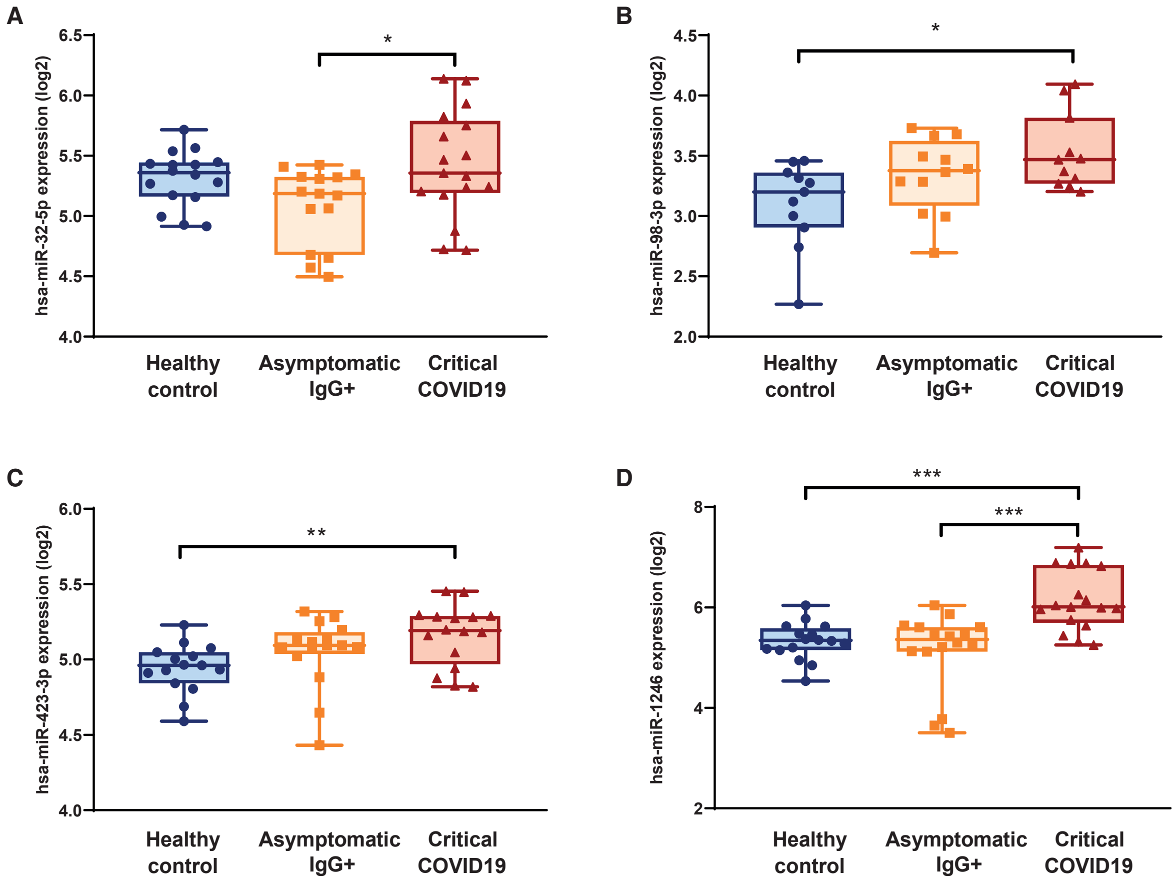

Figure 3. Serum miRNA levels, normalized to hsa-miR-103a-3p, in COVID-19-negative control subjects, asymptomatic IgG-positive donors, and critical COVID-19 patients

(A) hsa-miR-32-5p, (B) hsa-miR-98-3p, (C) hsa-miR-423-3p, and (D) hsa-miR-1246 serum levels. Data are presented in log2 with box and plot graphs representing the median, min, and max values, showing all data points. *$p < 0.05$, **$p < 0.01$, ***$p < 0.001$.

above, hsa-miR-1246 not only discriminated critical COVID-19 patients versus COVID-19-negative controls, it also allowed to distinguish critical versus asymptomatic patients, in agreement with a recent study in which has-miR-1246 appeared differentially expressed in severe versus asymptomatic COVID-19 patients, although this study was performed only with male patients.[46] Notablhashsa-miR-1246 has been described as a biomarker of emphysema in patients with chronic obstructive pulmonary disease,[47] and also with non-small cell lung cancer progression.[48] Moreover, miR-1246 has been identified as a possible regulator of the SARS-CoV-2 genome, which would provide more information on the protection mechanisms associated with miRNAs.[49] Concerning *ACE2* expression, a preliminary study described that the *ACE2* mRNA levels were inversely proportional to miR-1246 levels in the airways epithelium of smokers.[50] Noteworthy, in our study population most of our critical COVID-19 patients were non-smokers. Moreover, Khan and co-workers described, both *in silico* and *in vitro*, that *ACE2* was regulated by miR-1246 in patients with acute respiratory distress syndrome.[51,52] Finally, as regards *Rhas4*, hsa-miR-1246 has been also described as a potential prognostic biomarker for glioma, being predicted as one of its target genes.[53]

In addition to *ACE2* and *RAB14*, another 2,810 target genes were predicted for our 4 miRNAs. Overall, our *in silico* analysis indicated that these miRNAs are much more than simple post-transcriptional regulators since, besides, they have been involved in several biological processes related to the pathogenesis of SARS-CoV-2. For instance, the increase seen of these four circulating miRNAs may correlate, for example, with the decreased levels of *RAB14* transcripts found in lung biopsies from patients with adenocarcinomas,[54] together with many other crucial genes for SARS-CoV-2 infection. RAB14 participates in the formation of vesicles[55] necessary for the maturation and assembly of the structural proteins of SARS,[9,10] therefore it might also constitute an essential protein for infection by SARS-CoV-2.[56] In this sense, the analysis of the biological and functional roles indicated that not only *RAB14* but many other targeted genes appear involved in vesicular trafficking, cell-cell interactions, and regulation of the cell cycle, among others. In addition, these genes also participate in viral

Table 2. Comparisons of single and combined circulating miRNAs as predictors of critical COVID-19 patients

Groups	miRNA	AUC (95% CI)	Sensitivity (%)	Specificity (%)	p value
Critical COVID-19 versus COVID-19-negative control	hsa-miR-98-3p	0.8264 (0.6538–0.9991)	72.73	63.64	0.0095
	hsa-miR-423-3p	0.7875 (0.6199–0.9551)	75.00	73.33	0.0064
	hsa-miR-1246	0.8750 (0.755–0.995)	82.35	87.50	0.0002
	hsa-miR-98-3p + hsa-miR-423-3p + hsa-miR-1246	0.6634 (0.5484–0.7784)	63.64	69.05	0.0091
Critical COVID-19 versus asymptomatic IgG-positive COVID-19	hsa-miR-32-5p	0.7490 (0.5799–0.9181)	76.47	60.00	0.0165
	hsa-miR-1246	0.8824 (0.7681–0.9966)	82.35	87.50	0.0002
	hsa-miR-32-5p + hsa-miR-1246	0.7913 (0.6831–0.8994)	73.53	64.52	<0.0001

entry into the host cell, viral RNA replication, or even regulation of viral genome replication, which, together with the possible inhibition of the expression of RAB14 by the increase of the four miRNAs, could indicate a defense mechanism of the cells against the proliferation of the virus. Finally, several genes were also associated to regulation of the NLRP3 inflammasome complex, which has been linked to the severity of COVID-19,[57] and also with blood- and coagulation-related processes. This might correlate with the hypercoagulability and thrombotic events that take place in response of COVID-19.[58]

Conclusions

In this study, we have identified four miRNAs targeting *ACE2* and/or *RAB14* that could be taken as potential biomarkers of COVID-19 progression, allowing to distinguish critical patients from asymptomatic and negative individuals. The identified miRNA have been previously associated to respiratory-related diseases, including SARS-CoV. Moreover, many other gene targets of these miRNA have been associated with viral replication and inflammation- and coagulation-related processes. The individuals included in this study were recruited before being vaccinated, so any potential effect that vaccines might have over our results should be further evaluated. On the other hand, the limited access to serum samples in our study population constitutes a clear limitation in our study. Ideally, a higher number of samples should be analyzed to further validate the specificity of these miRNAs as biomarkers. Besides, even though peripheral miRNA levels were quantified, there was no confirmation about the direct secretion from the respiratory tissues into the extracellular space in COVID-19 patients, although previous research has shown their presence in the lungs. Future studies should include *in vitro* and *in vivo* models of COVID-19 to corroborate the bioinformatic predictions. These analyses might confirm the involvement of this four-miRNA panel as prognostic markers of SARS-CoV-2 infection, as well as their potential role as therapeutic candidates to inhibit the host response against this or other related viruses.

MATERIALS AND METHODS

Bioinformatic analysis to predict miRNAs that target ACE2, TMPRSS2, and RAB14

The miRDB database (http://mirdb.org/) and TargetScan (http://www.targetscan.org) were used to predict the miRNAs and their targeted genes,[19,59] and the network image was obtained using the miRNet (https://www.mirnet.ca) tool.[60] All these analyses were performed using the default parameters, and *Homo sapiens* was selected as the specific Taxonomy. The shared target genes of the predicted miRNAs were analyzed with STRING, an on-line platform to identify functional protein-association networks (https://string-db.org),[61] while the miRNA-gene network image was obtained with Flourish software (https://flourish.studio). Finally, STarMiR (www.sfold.wadsworth.org/cgi-bin/starmir.pl) was used for the prediction of miRNA binding sites to 3′ UTR mRNA binding sites (seeding region).

Study population

In total, 49 subjects were included in this study. Based on qPCR analysis against SARS-CoV-2, and ELISA tests for specific IgG and IgM antibodies (IME00136 and IME00137, Erba Mannheim), subjects were classified into three groups: (1) COVID-19-negative controls, which were PCR and IgG negative at the time of serum extraction (n = 16), (2) asymptomatic COVID-19 individuals, PCR negative and IgG positive at the time of serum extraction (n = 16), and (3) critical COVID-19 patients (n = 17) who required hospitalization. The first two groups (COVID-19-negative controls and asymptomatic donors) were enrolled at the National Paraplegic Hospital (Toledo, Spain), between April and May 2020. Critical COVID-19 patients were recruited at the time of hospitalization at the University Hospital Puerta del Mar (Cadiz, Spain) in July 2021, and the COVID-19 Hospital (Seville, Spain) in May 2021. The protocol was approved by the ethics committee at each center, and the study was conducted in accordance with the Helsinki II Declaration. Only donors older than 18 years were included in the study, and written informed consent was provided by all of those who participated in this study.

Serum collection

Peripheral blood samples were collected with serum separator tubes (SST II Advance, BD Vacutainer). The blood was then mixed up and down 8–10 times. After that, SST tubes were incubated at room temperature for at least 30 min to ensure the separation of the serum from the cellular components, and the serum was collected by centrifugation (2,000 × *g* for 10 min at 4°C). Serum samples were aliquoted and stored at −80°C until further use.

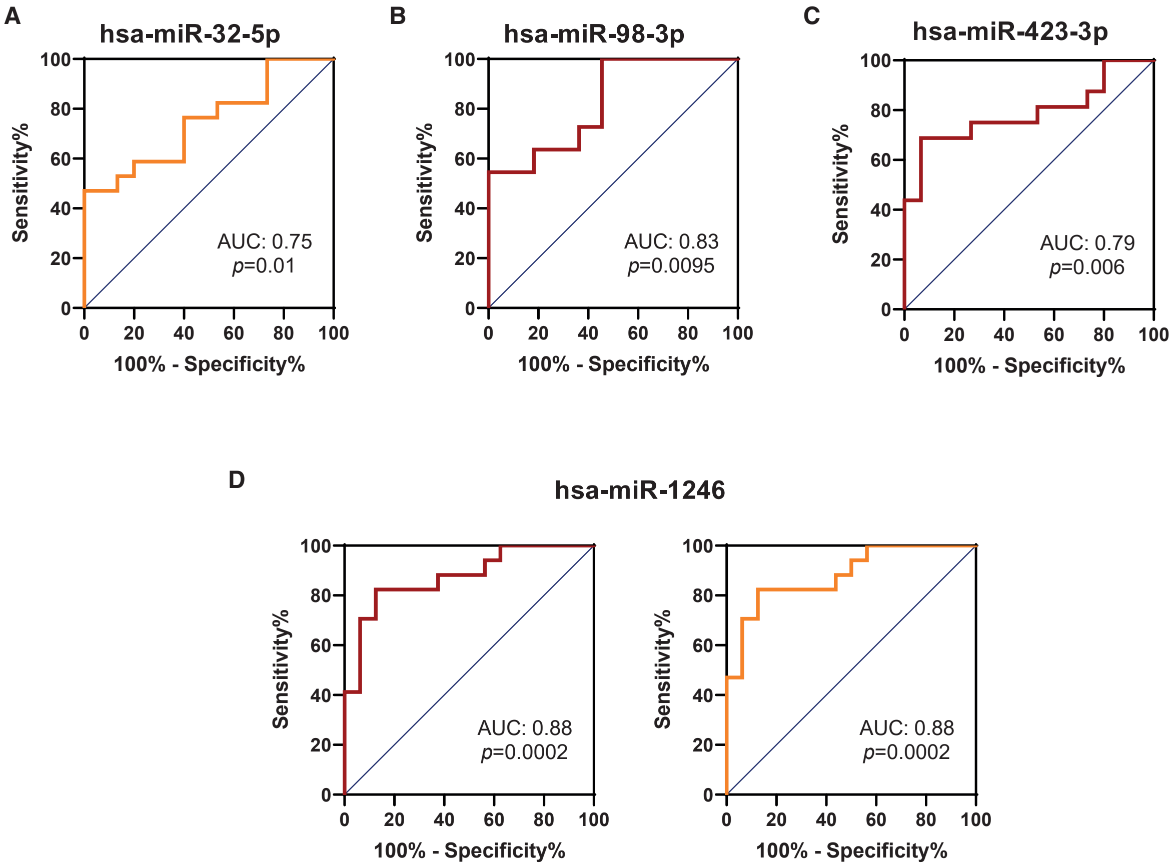

Figure 4. Receiver operating characteristic analysis of the miRNAs in critical COVID-19 patients with area under curve

(A) Receiver operating characteristic (ROC) analysis of hsa-miR-32-p in critical COVID-19 versus asymptomatic IgG-positive individuals. (B) ROC analysis of hsa-miR-98-3p in critical COVID-19 compared with COVID-19-negative controls. (C) ROC analysis of hsa-miR-423-3p in critical COVID-19 compared with COVID-19-negative controls. (D) ROC analysis of hsa-miR-1246 in critical COVID-19 compared with COVID-19-negative controls (left panel) and compared with asymptomatic IgG-positive subjects (right panel).

RNA isolation

Isolation of total RNA, including miRNAs, was performed with 200 μL of serum using the miRNeasy Serum/Plasma Kit (QIAGEN) following the manufacturer's instructions. Before purification, each serum sample was spiked (RNA spike-in, QIAGEN) with UniSp2 (2 fmol), UniSp4 (0.02 fmol), UniSp5 (0.00002 fmol), and MS2 RNA (Merck) to monitor the technical quality of RNA isolation according to the manufacturer's guidelines. Directly after isolation, RNA was subjected to the reverse transcription process.

Reverse transcriptase reaction

An MiRCURY LNA Reverse Transcription (RT) Kit (QIAGEN) was used to synthesize cDNA according to manufacturer's instructions. Isolated RNA (2 μL) were added to the reaction tube to make up a final volume of 10 μL reaction mix. UniSp6 (0.075 fmol) and cel-miR-39-3p (0.001 fmol) were used as positive controls for cDNA synthesis (QIAGEN). The reaction took place for 60 min at 42°C, heat inactivated for 5 min at 95°C, and immediately cooled to 4°C in a thermal cycler. Then cDNA samples were stored at −20°C.

Real-time quantitative polymerase chain reaction analysis of miRNAs expression levels

Samples from the RT reaction were prepared with the miRCURY SYBR Green PCR Kit (QIAGEN) and assessed for miRNA gene expression using the miRCURY LNA miRNA Serum/Plasma Focus PCR Panels (QIAGEN) according to the manufacturer's protocol. The interpolate calibrator UniSp3 was used to account for the variability between plates. Real-time qPCR analysis was performed in the CFX Connect PCR System (Bio-Rad) at 95°C for 2 min to heat samples, followed by 40 cycles of 95°C for 10 s, and 56°C for 60 s, followed by melting curve analysis. The analyzed miRNAs primer information can be found in Table S6. qPCR amplification curves were evaluated with CFX Manager software (Bio-Rad). The specificity of the amplification was confirmed by the melting curve analysis. Then, the expression level of each miRNA was calculated using the $2^{-\Delta Cq}$ method (where ΔCq = CqmiRNA − Cq hsa-miR-103a-3p). The normalized miRNA levels were further log2 converted.

Table 3. Correlation between the individual miRNAs levels and clinical variables in critical COVID-19 patients

	Hsa-miR-32-5p		Hsa-miR-98-3p		Hsa-miR-423-3p		Hsa-miR-1246	
	Pearson r	p	Pearson r	p	Pearson r	p	Pearson r	p
Age	−0.594	**0.006**	0.323	0.166	0.393	0.066	−0.226	0.192
Sex	0.047	0.429	−0.655	**0.014**	0.056	0.418	−0.111	0.335
COVID-19 severity	0.414	**0.049**	0.065	0.425	−0.035	0.449	0.219	0.199
Risk factor	−0.506	**0.019**	−0.437	0.090	0.296	0.132	−0.565	**0.009**

Functional enrichment analysis

Functional enrichment of predicted genes of differentially expressed miRNAs was analyzed using FunRich software (http://www.funrich.org/). The KEGG, GO, Uniprot, Reactome, and FunRich databases were used to identify the molecular functions and biological processes. Finally, the miRNA TissueAtlas (https://ccb-web.cs.uni-saarland.de/tissueatlas) was used to determine the expression of the selected miRNA within the tissues.

Statistical analysis

All statistical analyses were performed using GraphPad Prism 8 software. Comparisons among multiple groups were performed using one-way analysis of variance, followed by non-parametric Kruskal-Wallis rank tests. ROC curves were applied to characterize the diagnostic performance of both each and combined miRNAs. ROC curves were generated by plotting sensitivity against 100% specificity, indicating the AUC and 95% confidence intervals. Pearson correlation coefficient was used for correlations between log2 miRNAs versus clinical parameters in critical COVID-19 patients. Differences were considered statistically significant at $p < 0.05$.

SUPPLEMENTAL INFORMATION

Supplemental information can be found online at https://doi.org/10.1016/j.omtn.2022.06.006.

ACKNOWLEDGMENTS

We would like to thank the nurses, medical doctors, and other workers of the hospitals that contributed with the serum and data collection used in this study, especially to Carmen Rosell. Some images were obtained via SMART (https://smart.servier.com). This study was supported by GLOBALCAJA-Ayuda COVID-19 and Fondo Supera COVID-19, from Banco Santander and CRUE universidades, IPSA-COVID-19.

AUTHOR CONTRIBUTIONS

E.G.-B., M.P.M.-N., M.D.N.-M., T.T.-S., and M.A.R.-I., patients' recruitment and determination of patient infection by qRT-PCR. R.M.-L., M.D.N.-M., and M.A.R.-I. designed and managed the logistics of recruitment, collection, stratification, and sample storage. L.B.-C., S.E.-A., and M.R.-T. performed ELISA assays to confirm the infective stage (IgG/IgM). M.C.-D. and M.C.D.-R. conceived the

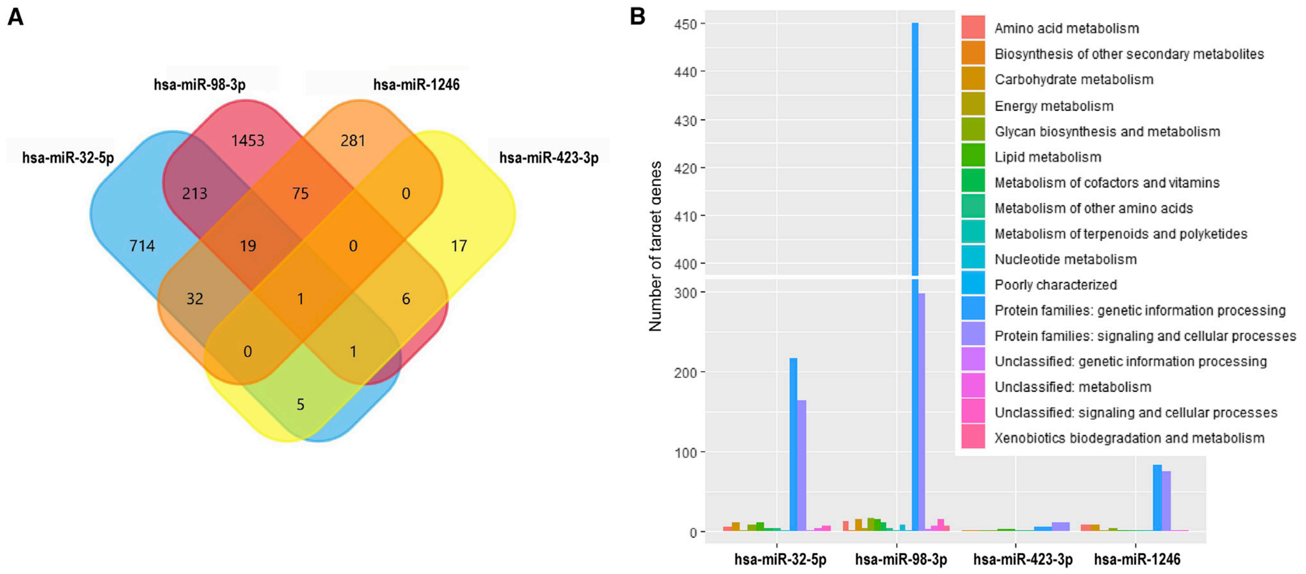

Figure 5. Target prediction of hsa-miR-32-5p, hsa-miR-98-3p, hsa-miR-423-3p, and hsa-miR-1246

(A) Venn diagram indicating the numbers of common and exclusive genes targeted by hsa-miR-32-5p, hsa-miR-98-3p, hsa-miR-423-3p, and hsa-miR-1246. B) KEGG categorization of targeted genes of hsa-miR-32-5p, hsa-miR-98-3p, hsa-miR-423-3p, and hsa-miR-1246; the x axis indicates KEGG categories, and the y axis indicates the numbers of gene targets.

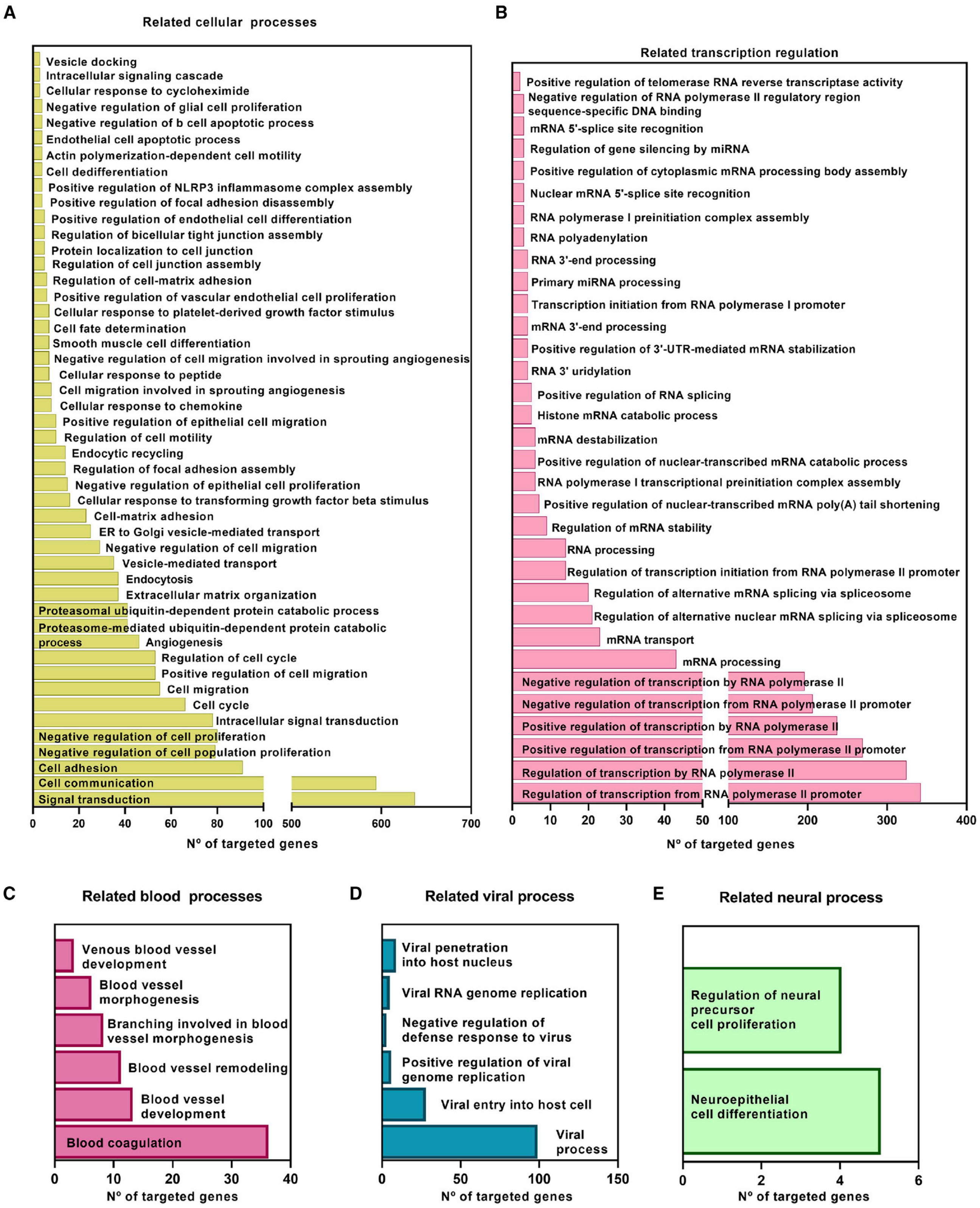

Figure 6. Functional enrichment analysis of hsa-miR-32-5p, hsa-miR-98-3p, hsa-miR-423-3p, and hsa-miR-1246 targeted genes
Bar plots representing functional enrichment analysis of cellular-related (A), transcriptional-related (B), blood-related (C), viral-related (D), and neural-related (E) processes.

experiments. M.C.-D., E.V.T., and A.G.-R. performed the miRNA sample analysis. M.C.-D., D.S.-M., and J.C.-D. performed the bioinformatic analysis. M.C.-D., E.T., and M.C.D.-R. contributed to manuscript writing. M.C.-D. and M.C.D.-R. evaluated the final data, and edited and revised the final manuscript. R.M.-L., J.A.M., and M.C.D.-R. conceptualized the project and revised the manuscript, providing final suggestions. All authors have read and approved the final manuscript.

DECLARATION OF INTEREST

The authors declare no competing interests.

REFERENCES

1. Cucinotta, D., and Vanelli, M. (2020). WHO declares COVID-19 a pandemic. Acta Biomed. *91*, 157–160. https://doi.org/10.23750/abm.v91i1.9397.
2. (2020). Listings of WHO's response to COVID-19. https://www.who.int/news/item/29-06-2020-covidtimeline.
3. (2020). Coronavirus disease (COVID-19) situation reports. https://www.who.int/emergencies/diseases/novel-coronavirus-2019/situation-reports.
4. Queen, D. (2022). Another year another variant: COVID 3.0—Omicron. Int. Wound J. *19*, 5. https://doi.org/10.1111/iwj.13739.
5. Fantini, J., Yahi, N., Colson, P., Chahinian, H., La Scola, B., and Raoult, D. (2022). The puzzling mutational landscape of the SARS-2-variant Omicron. Preprint at J. Med. Virol.. Online ahead of print.
6. Li, C., Hu, X., Li, L., and Li, J.H. (2020). Differential microRNA expression in the peripheral blood from human patients with COVID-19. J. Clin. Lab. Anal. *34*, e23590. https://doi.org/10.1002/jcla.23590.
7. Matarese, A., Gambardella, J., Sardu, C., and Santulli, G. (2020). miR-98 regulates TMPRSS2 expression in human endothelial cells: key implications for COVID-19. Biomed *8*, 462. https://doi.org/10.3390/biomedicines8110462.
8. Hoffmann, M., Kleine-Weber, H., Schroeder, S., Krüger, N., Herrler, T., Erichsen, S., Schiergens, T.S., Herrler, G., Wu, N.H., Nitsche, A., et al. (2020). SARS-CoV-2 cell entry depends on ACE2 and TMPRSS2 and is blocked by a clinically proven protease inhibitor. Cell *181*, 271–280.e8. https://doi.org/10.1016/j.cell.2020.02.052.
9. Gordon, D.E., Jang, G.M., Bouhaddou, M., Xu, J., Obernier, K., White, K.M., O'Meara, M.J., Rezelj, V.V., Guo, J.Z., Swaney, D.L., et al. (2020). A SARS-CoV-2 protein interaction map reveals targets for drug repurposing. Nature (Lond.) *583*, 459–468. https://doi.org/10.1038/s41586-020-2286-9.
10. Singh, M., Bansal, V., and Feschotte, C. (2020). A single-cell RNA expression map of human coronavirus entry factors. Cell Rep. *32*, 108175. https://doi.org/10.1016/j.celrep.2020.108175.
11. Papapanou, M., Papoutsi, E., Giannakas, T., and Katsaounou, P. (2021). Plitidepsin: mechanisms and clinical profile of a promising antiviral agent against COVID-19. J. Pers. Med. *11*, 668. https://doi.org/10.3390/jpm11070668.
12. White, K.M., Rosales, R., Yildiz, S., Kehrer, T., Miorin, L., Moreno, E., Jangra, S., Uccellini, M.B., Rathnasinghe, R., Coughlan, L., et al. (2021). Plitidepsin has potent preclinical efficacy against SARS-CoV-2 by targeting the host protein eEF1A. Science *371*, 926–931. https://doi.org/10.1126/science.abf4058.
13. Alam, T., and Lipovich, L. (2021). miRCOVID-19: potential targets of human miRNAs in SARS-CoV-2 for RNA-based drug discovery. Non-Coding RNA 7, 18.
14. Hum, C., Loiselle, J., Ahmed, N., Shaw, T.A., Toudic, C., and Pezacki, J.P. (2021). MicroRNA mimics or inhibitors as antiviral therapeutic approaches against COVID-19. Drugs *81*, 517–531. https://doi.org/10.1007/s40265-021-01474-5.
15. Mishra, R., Kumar, A., Ingle, H., and Kumar, H. (2019). The interplay between viral-derived miRNAs and host immunity during infection. Front. Immunol. *10*, 3079. https://doi.org/10.3389/fimmu.2019.03079.
16. Tang, H., Gao, Y., Li, Z., Miao, Y., Huang, Z., Liu, X., Xie, L., Li, H., Wen, W., Zheng, Y., and Su, W. (2020). The noncoding and coding transcriptional landscape of the peripheral immune response in patients with COVID-19. Clin. Transl. Med. *10*, e200. https://doi.org/10.1002/ctm2.200.
17. Guterres, A., de Azeredo Lima, C.H., Miranda, R.L., and Gadelha, M.R. (2020). What is the potential function of microRNAs as biomarkers and therapeutic targets in COVID-19? Infect. Genet. Evol. *85*, 104417. https://doi.org/10.1016/j.meegid.2020.104417.
18. Narożna, M., and Rubiś, B. (2021). Anti-SARS-CoV-2 strategies and the potential role of miRNA in the assessment of COVID-19 morbidity, recurrence, and therapy. Int. J. Mol. Sci. *22*, 8663. https://doi.org/10.3390/ijms22168663.
19. Agarwal, V., Bell, G.W., Nam, J.W., and Bartel, D.P. (2015). Predicting effective microRNA target sites in mammalian mRNAs. Elife *4*, e05005. https://doi.org/10.7554/elife.05005.
20. Dimmeler, S., and Nicotera, P. (2013). MicroRNAs in age-related diseases. EMBO Mol. Med. *5*, 180–190. https://doi.org/10.1002/emmm.201201986.
21. Sharma, S., and Eghbali, M. (2014). Influence of sex differences on microRNA gene regulation in disease. Biol. Sex Differ. *5*, 3. https://doi.org/10.1186/2042-6410-5-3.
22. WHO coronavirus (COVID-19) dashboard WHO coronavirus (COVID-19) dashboard with vaccination data. (2022) https://covid19.who.int/
23. Pereira, N.L., Ahmad, F., Byku, M., Cummins, N.W., Morris, A.A., Owens, A., Tuteja, S., and Cresci, S. (2021). COVID-19: understanding inter-individual variability and implications for precision medicine. Mayo Clin. Proc. *96*, 446–463. https://doi.org/10.1016/j.mayocp.2020.11.024.
24. Hu, B., Guo, H., Zhou, P., and Shi, Z.L. (2021). Characteristics of SARS-CoV-2 and COVID-19. Nat. Rev. Microbiol. *19*, 141–154. https://doi.org/10.1038/s41579-020-00459-7.
25. Donyavi, T., Bokharaei-Salim, F., Baghi, H.B., Khanaliha, K., Alaei Janat-Makan, M., Karimi, B., Sadri Nahand, J., Mirzaei, H., Khatami, A.R., Garshasbi, S., et al. (2021). Acute and post-acute phase of COVID-19: analyzing expression patterns of miRNA-29a-3p, 146a-3p, 155-5p, and let-7b-3p in PBMC. Int. Immunopharm. *97*, 107641. https://doi.org/10.1016/j.intimp.2021.107641.
26. Gao, M., Piernas, C., Astbury, N.M., Hippisley-Cox, J., O'Rahilly, S., Aveyard, P., and Jebb, S.A. (2021). Associations between body-mass index and COVID-19 severity in 6·9 million people in England: a prospective, community-based, cohort study. Lancet Diabetes Endocrinol. *9*, 350–359. https://doi.org/10.1016/s2213-8587(21)00089-9.
27. Kompaniyets, L., Goodman, A.B., Belay, B., Freedman, D.S., Sucosky, M.S., Lange, S.J., Gundlapalli, A.V., Boehmer, T.K., and Blanck, H.M. (2021). Body mass index and risk for COVID-19–related hospitalization, intensive care unit admission, invasive mechanical ventilation, and death — United States, march–december 2020. MMWR Morb. Mortal. Wkly. Rep. *70*, 355–361. https://doi.org/10.15585/mmwr.mm7010e4.
28. Leiva Sisnieguez, C.E., Espeche, W.G., and Salazar, M.R. (2020). Arterial hypertension and the risk of severity and mortality of COVID-19. Eur. Respir. J. *55*, 2001148. https://doi.org/10.1183/13993003.01148-2020.
29. Dennis, J.M., Mateen, B.A., Sonabend, R., Thomas, N.J., Patel, K.A., Hattersley, A.T., Denaxas, S., McGovern, A.P., and Vollmer, S.J. (2021). Type 2 diabetes and COVID-19–related mortality in the critical care setting: a national cohort study in england, march–july 2020. Diabetes Care *44*, 50–57. https://doi.org/10.2337/dc20-1444.
30. Lee, S.C., Son, K.J., Han, C.H., Jung, J.Y., and Park, S.C. (2020). Impact of comorbid asthma on severity of coronavirus disease (COVID-19). Sci. Rep. *10*, 21805. https://doi.org/10.1038/s41598-020-77791-8.
31. Sotiriou, S., Samara, A.A., Vamvakopoulou, D., Vamvakopoulos, K.O., Sidiropoulos, A., Vamvakopoulos, N., Janho, M.B., Gourgoulianis, K.I., and Boutlas, S. (2021). Susceptibility of β-thalassemia heterozygotes to COVID-19. J. Clin. Med. *10*, 3645. https://doi.org/10.3390/jcm10163645.
32. Van Seuningen, I., and Vincent, A. (2009). Mucins: a new family of epigenetic biomarkers in epithelial cancers. Expert Opin. Med. Diagn. *3*, 411–427. https://doi.org/10.1517/17530050902852697.
33. Zhang, J.X., Yang, W., Wu, J.Z., Zhou, C., Liu, S., Shi, H.B., and Zhou, W.Z. (2021). MicroRNA-32-5p inhibits epithelial-mesenchymal transition and metastasis in lung adenocarcinoma by targeting SMAD family 3. J. Cancer *12*, 2258–2267. https://doi.org/10.7150/jca.48387.
34. Wu, J., and Shen, Z. (2020). Exosomal miRNAs as biomarkers for diagnostic and prognostic in lung cancer. Cancer Med. *9*, 6909–6922. https://doi.org/10.1002/cam4.3379.

35. Hu, Y., Qin, X., Yan, D., Cao, H., Zhou, L., Fan, F., Zang, J., Ni, J., Xu, X., Sha, H., et al. (2017). Genome-wide profiling of micro-RNA expression in gefitinib-resistant human lung adenocarcinoma using microarray for the identification of miR-149-5p modulation. Tumor Biol. *39*. 101042831769165. https://doi.org/10.1177/1010428317691659.

36. He, B., Zhou, W., Rui, Y., Liu, L., Chen, B., and Su, X. (2021). Microrna-574-5p attenuates acute respiratory distress syndrome by targeting HMGB1. Am. J. Respir. Cell Mol. Biol. *64*, 196–207. https://doi.org/10.1165/rcmb.2020-0112oc.

37. Parzibut, G., Henket, M., Moermans, C., Struman, I., Louis, E., Malaise, M., Louis, R., Misset, B., Njock, M.S., and Guiot, J. (2021). A blood exosomal miRNA signature in acute respiratory distress syndrome. Front. Mol. Biosci. *8*, 640042. https://doi.org/10.3389/fmolb.2021.640042.

38. Sun, T., Yu, H., and Fu, J. (2020). Identification of key genes and miRNA-mRNA regulatory pathways in bronchopulmonary dysplasia in preterm infants by bioinformatics methods. Authorea Prepr. 1–12. https://doi.org/10.22541/au.159986212.28135679.

39. Zeng, J., Liu, W., Liang, J., Peng, J., Wang, F., Tang, J., Yang, Q., Zhuang, L., Huang, D., and Li, L. (2021). Analysis of miRNA profiles and the regulatory network in congenital pulmonary airway malformations. Front. Pediatr. *9*, 671107. https://doi.org/10.3389/fped.2021.671107.

40. Wu, F., Lu, F., Fan, X., Chao, J., Liu, C., Pan, Q., Sun, H., and Zhang, X. (2020). Immune-related miRNA-mRNA regulation network in the livers of DHAV-3-infected ducklings. BMC Genom. *21*, 123. https://doi.org/10.1186/s12864-020-6539-7.

41. Bilbao-Arribas, M., Abendaño, N., Varela-Martínez, E., Reina, R., de Andrés, D., and Jugo, B.M. (2019). Expression analysis of lung miRNAs responding to ovine VM virus infection by RNA-seq. BMC Genom. *20*, 62. https://doi.org/10.1186/s12864-018-5416-0.

42. Kumar, M., and Nerurkar, V.R. (2014). Integrated analysis of microRNAs and their disease related targets in the brain of mice infected with West Nile virus. Virology *452-453*, 143–151. https://doi.org/10.1016/j.virol.2014.01.004.

43. Farr, R.J., Rootes, C.L., Rowntree, L.C., Nguyen, T.H.O., Hensen, L., Kedzierski, L., Cheng, A.C., Kedzierska, K., Au, G.G., Marsh, G.A., et al. (2021). Altered microRNA expression in COVID-19 patients enables identification of SARS-CoV-2 infection. PLoS Pathog. *17*, e1009759. https://doi.org/10.1371/journal.ppat.1009759.

44. Zhu, Y., Li, T., Chen, G., Yan, G., Zhang, X., Wan, Y., Li, Q., Zhu, B., and Zhuo, W. (2017). Identification of a serum microRNA expression signature for detection of lung cancer, involving miR-23b, miR-221, miR-148b and miR-423-3p. Lung Cancer *114*, 6–11. https://doi.org/10.1016/j.lungcan.2017.10.002.

45. Vegh, P., Magee, D.A., Nalpas, N.C., Bryan, K., McCabe, M.S., Browne, J.A., Conlon, K.M., Gordon, S.V., Bradley, D.G., Machugh, D.E., and Lynn, D.J. (2015). MicroRNA profiling of the bovine alveolar macrophage response to Mycobacterium bovis infection suggests pathogen survival is enhanced by microRNA regulation of endocytosis and lysosome trafficking. Tuberculosis *95*, 60–67. https://doi.org/10.1016/j.tube.2014.10.011.

46. Parray, A., Mir, F.A., Doudin, A., Iskandarani, A., Danjuma, I.M.M., Kuni, R.A.T., Abdelmajid, A., Abdelhafez, I., Arif, R., Mulhim, M., et al. (2021). Snornas and mirnas networks underlying covid-19 disease severity. Vaccines *9*, 1056. https://doi.org/10.3390/vaccines9101056.

47. Cazorla-Rivero, S., Mura-Escorche, G., Gonzalvo-Hernández, F., Mayato, D., Córdoba-Lanús, E., and Casanova, C. (2020). Circulating miR-1246 in the progression of chronic obstructive pulmonary disease (COPD) in patients from the BODE cohort. Int. J. Chron. Obstruct. Pulmon. Dis. *15*, 2727–2737. https://doi.org/10.2147/copd.s271864.

48. Zhang, W.C., Chin, T.M., Yang, H., Nga, M.E., Lunny, D.P., Lim, E.K.H., Sun, L.L., Pang, Y.H., Leow, Y.N., Malusay, S.R.Y., et al. (2016). Tumour-initiating cell-specific miR-1246 and miR-1290 expression converge to promote non-small cell lung cancer progression. Nat. Commun. *7*, 11702. https://doi.org/10.1038/ncomms11702.

49. Chow, J.T.S., and Salmena, L. (2020). Prediction and analysis of SARS-CoV-2-targeting microrna in human lung epithelium. Genes *11*, 1002. https://doi.org/10.3390/genes11091002.

50. Zhang, H., Rostami, M.R., Leopold, P.L., Mezey, J.G., O'Beirne, S.L., Strulovici-Barel, Y., and Crystal, R.G. (2020). Expression of the SARS-CoV-2 ACE2 receptor in the human airway epithelium. Am. J. Respir. Crit. Care Med. *202*, 219–229. https://doi.org/10.1164/rccm.202003-0541oc.

51. Khan, A.T.A., Khalid, Z., Zahid, H., Yousaf, M.A., and Shakoori, A.R. (2022). A computational and bioinformatic analysis of ACE2: an elucidation of its dual role in COVID-19 pathology and finding its associated partners as potential therapeutic targets. J. Biomol. Struct. Dyn. *40*, 1813–1829. https://doi.org/10.1080/07391102.2020.1833760.

52. Liu, Q., Du, J., Yu, X., Xu, J., Huang, F., Li, X., Zhang, C., Li, X., Chang, J., Shang, D., et al. (2017). miRNA-200c-3p is crucial in acute respiratory distress syndrome. Cell Discov *3*, 17021. https://doi.org/10.1038/celldisc.2017.21.

53. Ji, B., Chen, L., Cai, Q., Guo, Q., Chen, Z., and He, D. (2020). Identification of an 8-miRNA signature as a potential prognostic biomarker for glioma. PeerJ *8*, e9943. https://doi.org/10.7717/peerj.9943.

54. Cotroneo, C.E., Mangano, N., Dragani, T.A., and Colombo, F. (2021). Lung expression of genes putatively involved in SARS-CoV-2 infection is modulated in cis by germline variants. Eur. J. Hum. Genet. *29*, 1019–1026. https://doi.org/10.1038/s41431-021-00831-y.

55. Spearman, P. (2018). Viral interactions with host cell Rab GTPases. Small GTPases *9*, 192–201. https://doi.org/10.1080/21541248.2017.1346552.

56. Hoffmann, H.-H., Sá Nchez-Rivera, F.J., Schneider, W.M., Macdonald, M.R., Poirier, J.T., and Rice, C.M. (2021). Functional interrogation of a SARS-CoV-2 host protein interactome identifies unique and shared coronavirus host factors. Cell Host Microbe *29*, 267–280.e5. https://doi.org/10.1016/j.chom.2020.12.009.

57. Rodrigues, T.S., de Sá, K.S.G., Ishimoto, A.Y., Becerra, A., Oliveira, S., Almeida, L., Gonçalves, A.V., Gonçalves, A.V., Perucello, D.B., Andrade, W.A., et al. (2021). Inflammasomes are activated in response to SARS-CoV-2 infection and are associated with COVID-19 severity in patients. J. Exp. Med. *218*, e20201707. https://doi.org/10.1084/jem.20201707.

58. Hanff, T.C., Mohareb, A.M., Giri, J., Cohen, J.B., and Chirinos, J.A. (2020). Thrombosis in COVID-19. Am. J. Hematol. *95*, 1578–1589. https://doi.org/10.1002/ajh.25982.

59. Chen, Y., and Wang, X. (2020). miRDB: an online database for prediction of functional microRNA targets. Nucleic Acids Res. *48*, D127–D131. https://doi.org/10.1093/nar/gkz757.

60. Chang, L., Zhou, G., Soufan, O., and Xia, J. (2020). miRNet 2.0: network-based visual analytics for miRNA functional analysis and systems biology. Nucleic Acids Res. *48*, W244–W251. https://doi.org/10.1093/nar/gkaa467.

61. Szklarczyk, D., Gable, A.L., Nastou, K.C., Lyon, D., Kirsch, R., Pyysalo, S., Doncheva, N.T., Legeay, M., Fang, T., Bork, P., et al. (2021). The STRING database in 2021: customizable protein–protein networks, and functional characterization of user-uploaded gene/measurement sets. Nucleic Acids Res. *49*, D605–D612. https://doi.org/10.1093/nar/gkaa1074.

OMTN, Volume *29*

Supplemental information

Serum microRNAs targeting *ACE2* and *RAB14* genes distinguish asymptomatic from critical COVID-19 patients

Maria Calderon-Dominguez, Eva Trejo-Gutierrez, Almudena González-Rovira, Lucía Beltrán-Camacho, Marta Rojas-Torres, Sara Eslava-Alcón, Daniel Sanchez-Morillo, Juan Calderon-Dominguez, Ma Pilar Martinez-Nicolás, Estibaliz Gonzalez-Beitia, Ma Dolores Nieto-Martín, Teresa Trujillo-Soto, Manuel A. Rodríguez-Iglesias, Juan A. Moreno, Rafael Moreno-Luna, and Ma Carmen Durán-Ruiz

Table S1. Bibliographic references of miRNAs related to the respiratory system

miRNAs	DOI or PMID
miR-32-5p	10.1517/17530050902852697<br>10.7150/jca.48387<br>10.1186/s12864-018-5416-0<br>10.1165/rcmb.2020-0112OC<br>10.3892/mmr.2021.11851<br>10.3389/fped.2021.671107
miR-98-3p	10.1177/1010428317691659<br>10.1002/CAM4.3379<br>10.3389/fmolb.2021.640042<br>10.1002/jcb.29612
miR-214-3p	10.1161/CIRCRESAHA.119.315067<br>10.1164/rccm.201807-1237OC<br>10.12659/MSM.915709<br>10.1038/s41389-019-0151-1<br>10.1089/ars.2019.7965
miR-421	10.1159/000503020<br>10.3892/ol.2020.12169<br>10.2147/CMAR.S167432<br>10.26355/eurrev_201905_17805<br>10.2147/CMAR.S258887<br>10.1016/j.prp.2019.152555<br>10.1042/CS20130420<br>10.1158/1055-9965.EPI-15-0161<br>10.1002/jcb.28945<br>10.2147/CMAR.S248869
miR-423-3p	10.1016/j.tube.2014.10.011<br>10.1186/s12920-020-00748-3<br>10.14336/AD.2015.0620<br>10.1186/s13000-019-0831-3<br>10.1016/j.lungcan.2017.10.002<br>10.1186/s11658-021-00247-y<br>10.3390/ijms21155381
miR-1246	10.1080/07391102.2020.1833760<br>10.4143/crt.2018.638<br>10.1164/rccm.202003-0541OC<br>10.7717/peerj.9943<br>10.1016/j.lungcan.2015.11.013<br>10.2147/JAA.S331090<br>PMID: 28386354

Table S2. The binding site position of candidate miRNAs at 3’UTR mRNA

3’UTR mRNA	miRNA name	Number of binding sites	Position in 3´UTR sequence (canonical matched sites)
ACE2	hsa-miR-421	3	2567-2591, 2567-2587, 2488-2509
	hsa-miR-1246	3	1285-1317, 450-468, 2397-2433
TMPRSS2	hsa-miR-214-3p	5	1880-1905, 1942-1962, 1770-1798, 2422-2438, 2340-2355
RAB14	hsa-miR-32-5p	2	2822-2843, 1853-1870
	hsa-miR-98-3p	6	1527-1543, 3087-3093, 1578-1583, 1941-1947, 2500-2537, 2237-2269
	hsa-miR-214-3p	6	1824-1845, 2419-2439, 2027-2056, 2719-2744, 2877-2914, 1855-1890
	hsa-miR-423-3p	1	1161-1175
	hsa-miR-1246	3	2666-2692, 3382-3407, 1423-1456

Table S3. Clinical variables of the study population

	COVID-19 negative control N=16	Asymptomatic IgG+ COVID-19 patients N=16	Critical COVID-19 patients N=17
Age (years)	50±2.18	49.31±1.99	44.88±4.35
Sex (male)	6.3 %	31.3 %	35.5 %
Symptom (%)	12.2 %	50 %	100 %
Positive qRT-PCR.COVID-19 (%)	0 %	0 %	100 %
Positive IgG	0 %	100 %	0 %
Risk factor	12.5 %	18.8 %	52.92 %
Obesity	0 %	12.5 %	23.52 %
Dyslipidemia	6.3 %	0 %	11.75 %
Type 2 Diabetes	0 %	0 %	5.88 %
Asthma	6.3 %	0 %	5.9 %
Arterial hypertension	6.3 %	6.3 %	23.52 %
Smoker	25 %	18.75 %	5.88 %

All values are expressed as mean ± SEM

Table S4. Functional enrichment analysis based on based on hsa-miR-32-5p, hsa-miR-98-3p, hsa-miR-423-3p and hsa-miR-1246 target genes. This Table is included as an excel file.

Table S5. miRNA expression data in COVID-19 samples compared to other respiratory disorders.

miRNAs	Expression in our Critical COVID-19 patients	Expression in other respiratory disorders	Sample	DOI or PMID
**hsa-miR-32-5p**	Upregulated	Downregulated (*p*=0.01) (Figure R2.Q2.B)	NSCLC tissues	10.7150/jca.48387
		Downregulated (*p*<0.05) (Figure R2.Q2.A)	Plasma from ARDS patients	10.1165/rcmb.2020-0112OC
		Upregulated (FC=4.9954; *p*=0.003)	Peripheral blood from BPD patients	10.1155/2018/6204128
		Downregulated (FC= 8.09; *p*= 0.006) Data available in the Supplementary information	Lung tissues from CPAMs patients	10.3389/fped.2021.671107
**miR-98-3p**	Upregulated	Upregulated (FC=3.8078613; *p*=0.009)	NSCLC cell line	10.1177/1010428317691659
		Downregulated (FC=0.12; *p*=0.022)	Plasma from LAC patients	10.1002/jcb.29612
		Upregulated (FC= 20.64; *p*=0.0091)	Plasma from ARDS patients	10.3389/fmolb.2021.640042
**hsa-miR-423-3p**	Upregulated	Upregulated (FC= 5.95; *p*<0.05)	Serum from LC patients	10.1016/j.lungcan.2017.10.002
**hsa-miR-1246**	Upregulated	Downregulated (FC= 1.46; *p*=0.027)	Serum from COPD patients	10.2147/COPD.S271864
		Upregulated (*p*<0.05) (Figure R2.Q2.C and D)	NSCLC tissues and LUAD tissues	10.1038/ncomms11702

Abbreviations: **ARDS,** acute respiratory distress syndrome; **BPD**, bronchopulmonary dysplasia; **COPD,** chronic obstructive pulmonary disease; **CPAMs**, congenital pulmonary airway malformations; **FC**, fold change; **LAC**, lung adenocarcinoma; **LC**, lung cancer**; LUAD**, lung squamous carcinoma; **NSCLC**, Non-small cell lung cancer

Table S6. Analyzed miRNAs and their manufacturer's information

miRNA ID	Product Name	GeneGlobe Id	Catalog Number	Accession number	Sequence
hsa-miR-32-5p	hsa-miR-32-5p miRCURY LNA miRNA PCR Assay	YP00204792	339306	MIMAT0000090	5'UAUUGCACAUUACUAAGUUGCA
hsa-miR-98-3p	hsa-miR-98-3p miRCURY LNA miRNA PCR	YP02106353	339306	MIMAT0022842	5'CUAUACAACUUACUACUUUCCC
hsa-miR-214-3p	hsa-miR-214-3p miRCURY LNA miRNA PCR Assay	YP00204510	339306	MIMAT0000271	5'ACAGCAGGCACAGACAGGCAGU
hsa-miR-421	hsa-miR-421 miRCURY LNA miRNA PCR Assay	YP00204603	339306	MIMAT0003339	5'AUCAACAGACAUUAAUUGGGCGC
hsa-miR-423-3p	hsa-miR-423-3p miRCURY LNA miRNA PCR Assay	YP00204488	339306	MIMAT0001340	5'AGCUCGGUCUGAGGCCCCUCAGU
hsa-miR-1246	hsa-miR-1246 miRCURY LNA miRNA PCR Assay	YP00205630	339306	MIMAT0005898	5'AAUGGAUUUUUGGAGCAGG
hsa-miR-103a-3p	hsa-miR-103a-3p miRCURY LNA miRNA PCR Assay	YP00204063	339306	MIMAT0000101	5'AGCAGCAUUGUACAGGGCUAUGA

Figure S1

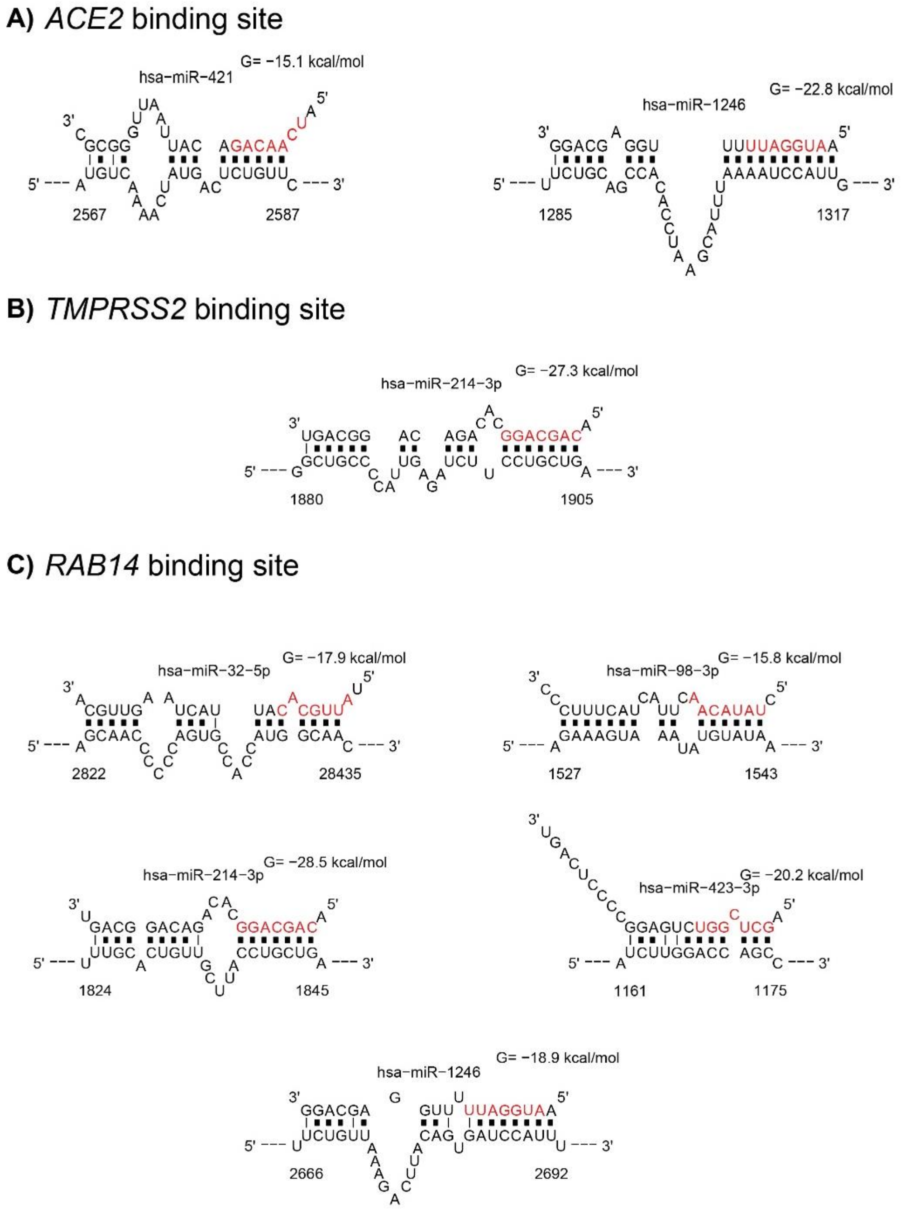

Figure S1. Predicted binding site of selected miRNAs in the ACE2, TMPRSS2, and RAB14 3′-UTR. The figure summarizes the information provided by STarMir and RNA hybrid on location, sequence complementarity, free energy, and target site accessibility. ACE2, TMPRSS2, and RAB14 3′-UTR sequences are written in black, whereas the selected microRNA seed regions appear in red. A) Binding site of hsa-miR-421 and hsa -miR-1246 in 3'UTR of ACE2 mRNA. B) Binding site of hsa-miR-214-3p in 3'UTR of TMPRSS2. C) Binding site of hsa-miR-32-5p, hsa-miR-98-3p, hsa-miR-214-3p, hsa-miR-423-3p, and hsa-miR-1246 in 3'UTR of ACE2 mRNA.

Figure S2

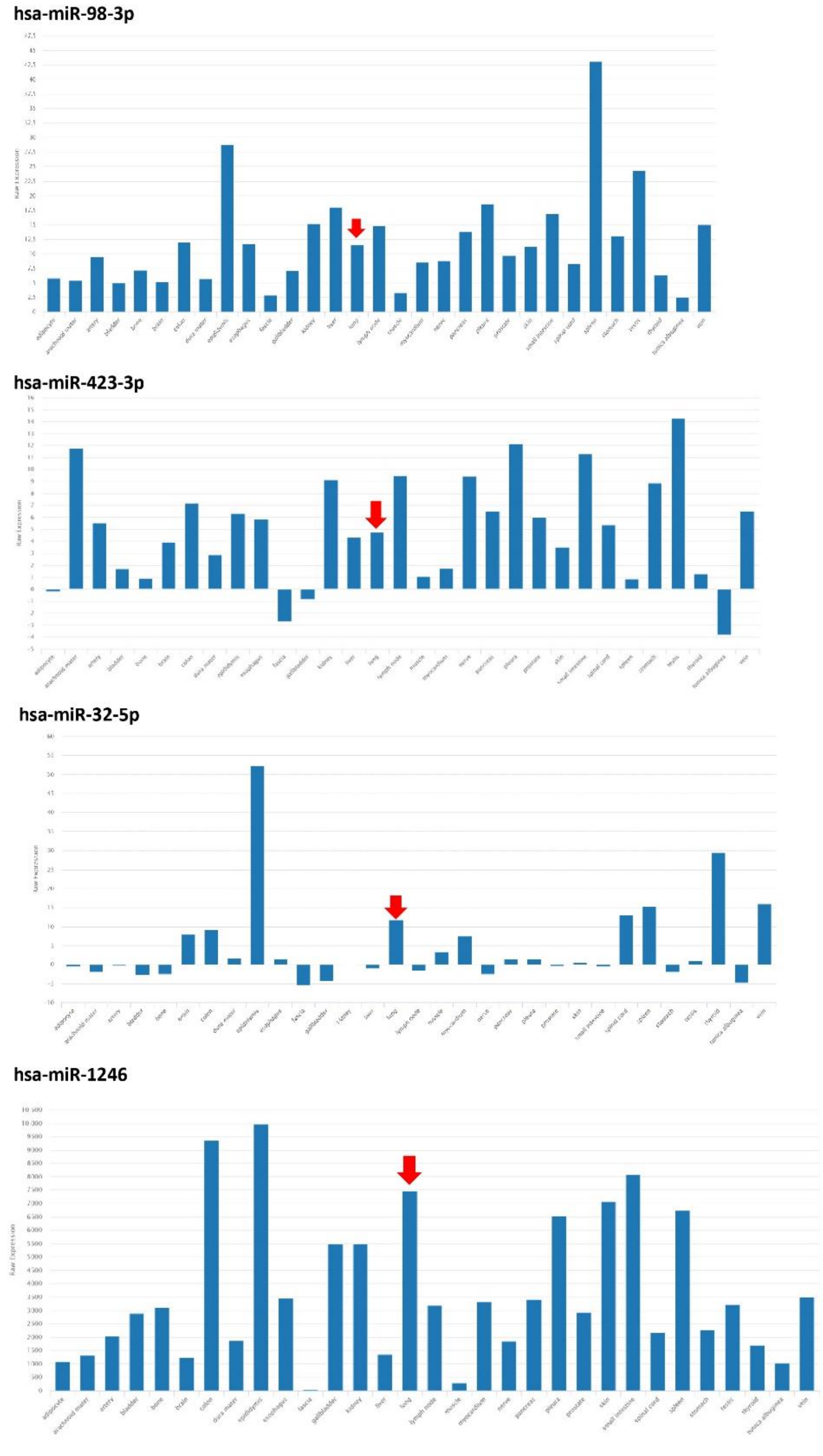

Figure S2. miRNAs normalized expression across organ systems. The figure summarizes the information provided by the Human miRNA Tissue atlas (https://ccb-web.cs.uni-saarland.de/tissueatlas). The red flag indicated the predicted expression of hsa-miR-98-3p, hsa-miR-32-5p, hsa-miR-423-3p, and hsa-miR-1246 in the lungs.

Figure S3

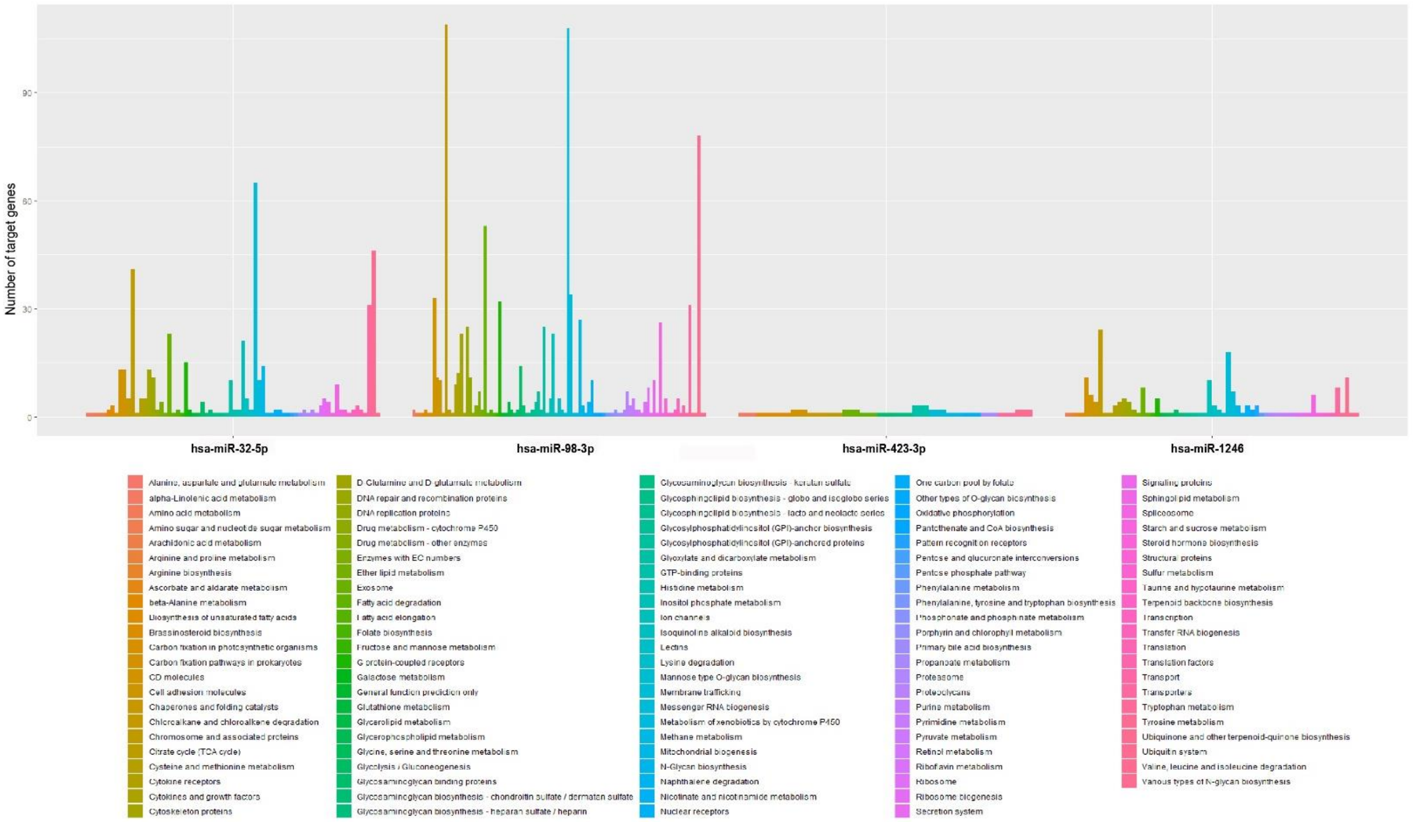

Figure S3. Extended KEGG categorization of targeted genes of hsa-miR-32-5p, hsa-miR-98-3p, hsa-miR-423-3p, and hsa-miR-1246. The x-axis indicates KEGG categories, and the y-axis indicates the number of gene targets.
